# Integration of NanoMIPs With Gold‐Patterned Plasmonic Surfaces as Synthetic Protein A

**DOI:** 10.1002/smsc.70394

**Published:** 2026-09-02

**Authors:** Fabiana Grillo, Francesco Gentile, Francesco Canfarotta, Maria Laura Coluccio, Elena Piletska, Donald Jones, Thong H. Cao, Virginia Garo, Nilde Fera, Sandro Dragone, Patrizio Caldeloro, Sergey Piletsky, Natalia Malara

**Affiliations:** ^1^ Department of Chemistry University of Leicester Leicester UK; ^2^ Department of Experimental and Clinical Medicine, BioNEM Lab University Magna Graecia Catanzaro Italy; ^3^ Tozaro Ltd. Sharnbrook UK; ^4^ School of Chemistry, College of Science and Engineering University of Leicester Leicester UK; ^5^ Department of Cancer Studies, RKCSB University of Leicester Leicester UK; ^6^ Department of Cardiovascular Sciences University of Leicester Leicester UK; ^7^ Department of Health Sciences University Magna Graecia Catanzaro Italy; ^8^ Department of Medical and Surgical Sciences University Magna Graecia Catanzaro Italy

**Keywords:** gold nanoparticles, IgG, metal‐enhanced fluorescence, nanoMIPs, protein

## Abstract

In modern bioanalytical sensing, the integration of biorecognition elements with transducer platforms represents an established strategy for achieving selective detection. Among the available bioreceptors, proteins have been widely used for their ability to mediate highly specific interactions. However, proteins often present limitations such as instability, high production costs, animal‐derived manufacturing, and strict storage protocols. A promising alternative is molecularly imprinted polymeric nanoparticles (nanoMIPs), which have shown potential due to features such as greater stability, durability, and potentially lower costs. This study introduces, for the first time, the use of nanoMIPs imprinted for IgG on a silicon surface functionalised with gold nanoparticles. These nanoMIPs are designed to bind IgG in an oriented manner by targeting Fc‐specific epitopes selected through a novel epitope‐mapping technique. Detection is based on metal‐enhanced fluorescence (MEF), which significantly amplifies the signal. The sensor showed high specificity for IgG, with no cross‐reactivity toward IgA, reaching a detection limit of 10 ng mL^−1^. Compared with protein A, the natural binder of IgG, nanoMIPs achieved superior performance by 20.6% and 17.8% for the two epitopes tested.

## Graphical abstract

Epitope‐imprinted nanoMIPs are combined with plasmonic metal‐enhanced fluorescence to create a synthetic protein A‐like sensing platform for human IgG. The approach enables oriented antibody recognition with high sensitivity and selectivity, providing a robust route toward miniaturised and multiplexed diagnostic devices.

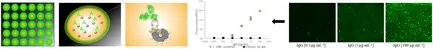

## 1 Introduction

In the past 20 years, interest in integrating bio‐recognition elements with sensor platforms has grown substantially [1]. The need for specific and accurate recognition of target molecules in complex matrices has led to a significant demand and growth in the development of reliable and robust detection platforms. Antibodies represent the most used affinity reagent for a variety of different applications [2]. ELISA is nowadays an established tool for In Vitro Diagnostics (IVD), widely used in the initial screening, treatment monitoring, and prognosis evaluation of various diseases. However, some issues such as poor stability and the need for specific storage conditions resulted in limited applicability onsite. In addition, even though the researchers have developed an increasing variety of recombinant production systems for the expression of antibodies (e.g. bacteria, yeasts, mammalian cell lines, and transgenic plants and animals), post‐translational modification still causes issues in antibody expression, resulting in the indispensable use of animals for their expression. All this led to a subsequent overall increase in production costs and therefore price for the customers.

**FIGURE 1:**
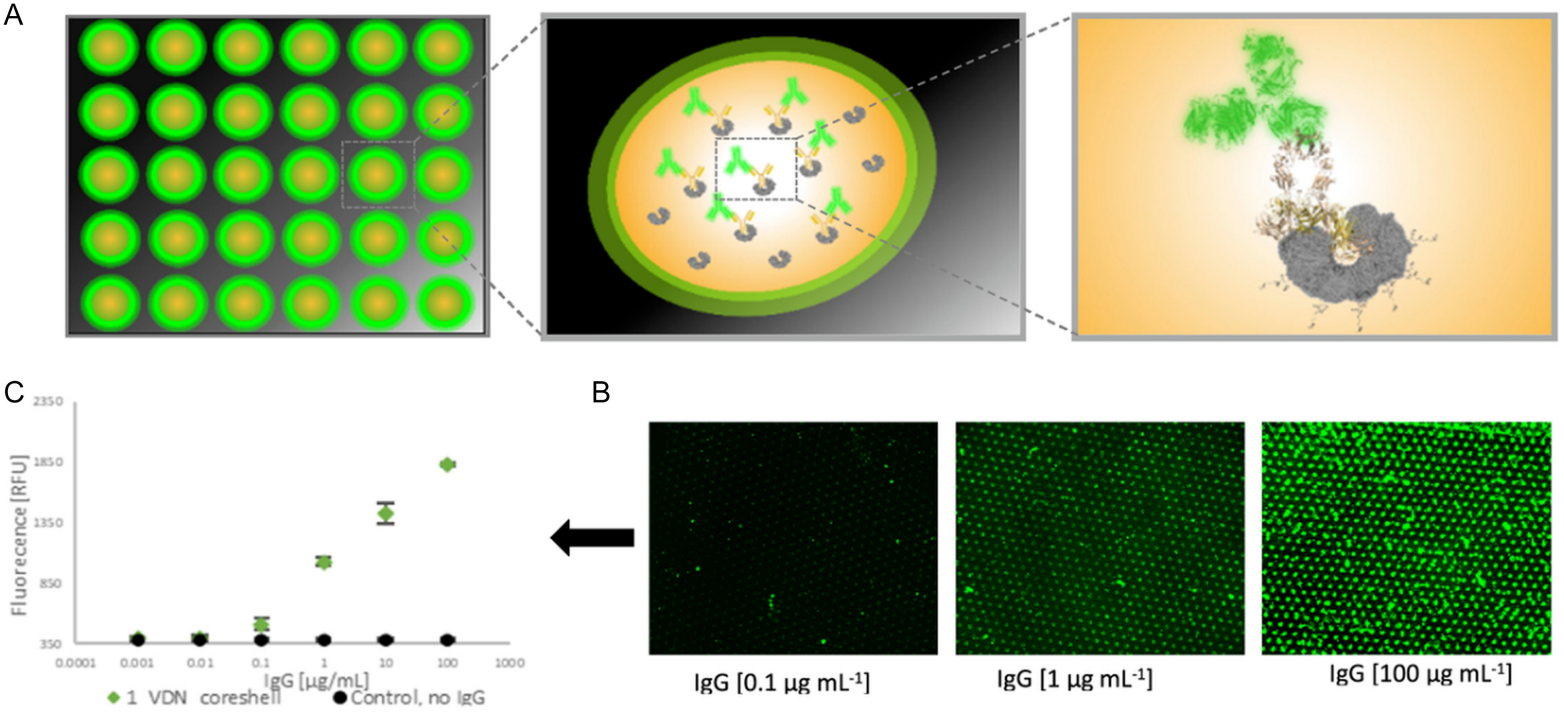
Schematic representation of the gold‐patterned plasmonic platform functionalisation, nanoMIPs immobilised on gold clusters binding IgG, fluorescent IgG (A); microscope images of IgG dilutions (B); graph derived from analysis (C).

In recent years, molecularly imprinted polymers (MIPs) have demonstrated the efficient recognition of molecules with different structural properties, such as small molecules, peptides, proteins, and whole cells [3]. Their versatility and robustness in harsh conditions make them a potential candidate for point‐of‐care detection [4]. MIPs have demonstrated to be a suitable synthetic replacement for antibodies and therefore, not surprisingly, abundant literature has been generated on MIPs in assays and sensor platforms [5]. The overall concept of the nanoMIP‐based plasmonic platform is illustrated in Figure 1.

**FIGURE 2:**
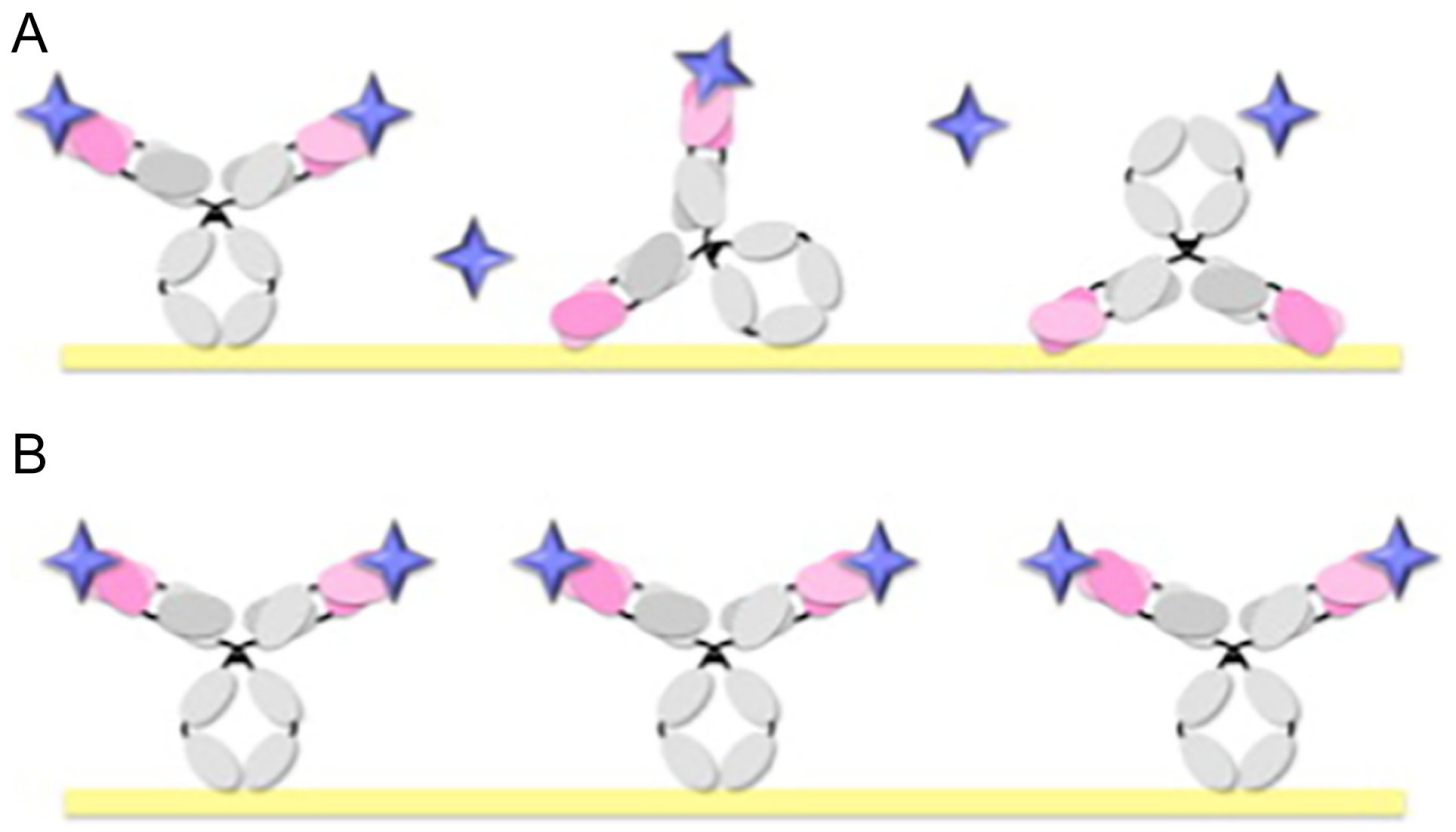
Schematic representation of two main approaches to antibody immobilisation. Random (A) and site‐directed antibody immobilisation (B). Image reproduced with permission from [7].

In most protein assays, arrays, or substrates, the target protein must first encounter the affinity reagent immobilised on the surface, and their performance is highly dependent on the interaction between the binding site availability of the affinity reagent and its target analyte [6] (Figure 2). To date, several studies have demonstrated the crucial role of the efficiency of the immobilisation plays of the binding reagent to a given surface in the interaction with the target analyte. Several different strategies are present in the literature, such as covalent immobilisation, biotin‐streptavidin mediated interactions, and the use of chemical modification of the surface with affinity reagents [8]. In addition to the immobilisation strategies, the orientation of the binding site turns out to be a key factor, whereby maximising its exposure to the target analyte results in an overall increased efficiency of binding [9]. Similarly to the immobilisation strategies, the team have used similar approaches to facilitate the orientation of the binding site, with some of the most efficient strategies being the use of aid proteins aimed at the binding to the Fc regions of antibodies (e.g. protein A and G) [10].

Protein A was originally utilised in the biotechnology industry for purification purposes, with its first application in 1976 in the form of a chromatography resin [11]. Since then, a multitude of other applications were explored. The demand for antibody purification initially drove protein A’s application in separations towards resins [12]; however, considering the range of applications it was soon after adapted to, its potential in orienting antibodies for diagnostics purposes became evident. Nowadays, protein A is typically functionalised with metallic beads (gold or iron oxide nanoparticles (IO‐NPs) for signalling or separation aims [13, 14], coupled to fluorescent dyes for indirect ELISAs or coated to 96‐well plates to orient Ig immobilisation in sandwich ELISAs [15]. The already established protein A market is expected to rise from USD 1.1 billion in 2020 and reach USD 1.9 billion by 2025 [16]. Over the years, many efforts were made in optimising the production process, however, the high cost still represents a limiting factor. Being able to efficiently replace protein A with a synthetic counterpart, would provide a significant advancement to the diagnostic and separation field. Molecularly imprinted polymers have been demonstrated to be an efficient replacement to bio‐recognition elements and cases of MIPs synthesised for the extraction or detection of IgG are reported in the literature [17, 18, 19, 20, 21]. However, in these cases, the imprinting was directed to the whole protein with no directionality to the site of binding. Two different strategies were recently adopted to orient the imprinting. In the work conducted by Çorman et al. [22] MIPs were imprinted for an L‐lysine peptide typically found at the C‐terminus of the Fc region of antibodies. However, this is a motif shared between different classes of IgGs; therefore, the selectivity of the resulting MIPs is not guaranteed [23]. Very recently, Moczko et al. [24] instead imprinted nanoMIPs against the whole IgG, Fc region, and a single peptide from the constant Fc region and proved that selectively imprinting one region of the antibody is beneficial to the binding. Selectively identifying exposed epitopes on IgG might cooperate to orient the binding and produce high affinity and site–site specific MIPs able to detect or orient IgG position as synthetic protein A.

Biomedical diagnostics are leaning towards greater miniaturisation and portability, aiming at maintaining precision and enhanced performance of the devices [25, 26]. Increasing accuracy and detection limit of the instruments will allow the use of smaller sample volumes and less invasive sampling methodologies with consequent minimal distress for the patient. Some devices, with high precision and small surface and volume for the detection of biological molecules, are already present on the market [27, 28]. Such surfaces are usually patterned with detection areas ranging from 100 μm to 2 mm in diameter [29]. Reducing the surface needed for detection increases space availability for multiplexing the detection of several analytes in the same miniaturised device. Reducing the number and volume of samples, again, concurs with the overall aim of causing the least distress to the patient.

**FIGURE 3:**
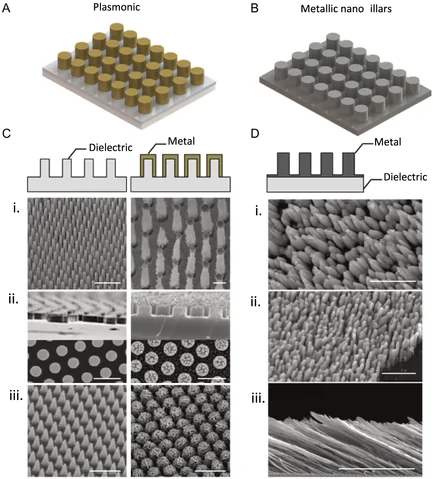
Schematic illustration of plasmonic dielectric nanopillar array and metallic nanopillar arrays (A,B). Plasmonic dielectric nanopillars cover metal nanostructures on dielectric nanopillar arrays (A). Several engineering methods for plasmonic dielectric nanopillar array and metallic nanopillar array (C,D); tapered silver nanopillar array made by laser interference lithography before (left) and after metal deposition (right) (scale bar: 1 μm (right) and 200 nm (left)) (C‐i). Silver nanofilm annealed on top of the silicon nanopillar array with the assistance of nanosphere lithography before (left) and after silver film deposition and following annealing process (right) (scale bar: 500 nm) (C‐ii). Silver nanoplate hierarchical turreted and ordered arrays (HTOAs) fabricated through an electro‐deposition method on ordered silicon nanocones with the assistance of nanosphere lithography and reactive ion etching before (left) and after formation of 3D silver nanoplate HTOAs (right) (scale bar: 2 μm) (C‐iii). Silver nanoneedles fabricated by ion beam irradiation on tilted substrates (scale bar: 1 μm) (D‐i). Vertically aligned, single crystalline silver nanorods on silicon substrate via the glancing angle deposition with electron beam evaporator (scale bar: 500 nm) (D‐ii). Silver nanorod array using an oblique angle deposition technique on glass slides at low substrate temperature (scale bar: 2 μm) (D‐iii). Figure reproduced with permission from [30].

In recent years, platforms with metal spots, particularly nanopillar arrays, have become increasingly popular due to their highly controlled structure, adaptable in various applications [30]. For instance, nanopillar arrays‐based surfaces can induce cell differentiation [31, 32, 33], show a controlled hydrophobicity and consequent droplets manipulation [34, 35], but also produce localised electromagnetic fields. Multidimensional structures of nanopillar arrays are particularly beneficial for providing well‐designed hot spots with high density and for improving fluorescence signals [36]. Their physical characteristics are beneficial for surface‐enhanced Raman spectroscopy/scattering (SERS), a technique that has gained much attention in recent years due to its high sensitivity in diagnostic applications [37], allowing to detect compounds even at the single molecule level [38, 39]. Over time, different strategies for the fabrication of nanopillars have been explored, as carefully reviewed by Oh et al. [30] Figure 3 summarises some of the most successful strategies used [30].

Battista et al. [40] have reported a nanofabricated plasmonic platform patterned with gold nanoclusters fabricated via optical lithography and electroless deposition. The two‐step separation allowed the realisation of a device where size and spacing of the silicon pillars and the gold particles were separately controlled at the nano level in a 3D architecture. In this work, metal‐enhanced fluorescence (MEF) was applied. MEF is a technique extensively explored in the past 10 years [40, 41, 42] that is generated by surface plasmon resonance (SPR), i.e. the collective oscillations of electrons on the surface of a gold nanomaterial. SPR can result in subwavelength light confinement and enhancement near the metallic surface [43, 44, 45]. In MEF, fluorescence amplification depends on three separate mechanisms: (1) energy transfer from the fluorophore to the metal, (2) enhancement of the local electromagnetic field, and (3) modification of the radiative decay rate of the fluorophore through the local modification of the photon density of states [41]. In the device reported by Battista et al., the hierarchical structure of gold nanoparticles arrays demonstrated to enhance the fluorescence efficiency compared to untreated planar substrates. Following this work, a plasmonic platform fabricated in the same manner was utilised as biosensor for point‐of‐care analysis of immunoglobulins in urine, where IgG was detected with a fluorescently labelled anti‐IgG, showing a detection limit of 10 µg L^−1^ [46]. Numerous examples of MEF integrated in different biosensor platforms are present in the literature [47], mostly characterised by the use of antibodies as recognition elements. To date, no examples in the literature were reported on nanoMIPs integration in plasmonic platforms patterned with gold nanoclusters. This research work aimed at the integration of nanoMIPs with a plasmonic platforms for the oriented binding of specific epitopes of IgG.

In this work, we propose a plasmonic sensing platform based on nanoMIPs for the selective and oriented detection of human IgG. The aim is to provide a more stable and robust synthetic alternative to traditional protein‐based affinity reagents, such as protein A, which are often limited by high costs, instability, and strict storage requirements. Unlike systems imprinted against the whole protein, the nanoMIPs developed in this study were designed using specific epitopes from the Fc region of IgG, selected through a MIP‐based epitope discovery approach supported by structural and sequence analyses. This strategy made it possible to identify exposed regions of the molecule, promoting a more targeted and directional recognition of IgG. The selected nanoMIPs were then immobilised, for the first time, onto a silicon platform decorated with gold nanoclusters, which enhances the fluorescent signal through metal‐enhanced fluorescence. The study included epitope selection, integration of the nanoMIPs onto the plasmonic surface, optimisation of the fluorescent signal, evaluation of the response to different IgG concentrations, tests in complex protein mixtures, cross‐reactivity assessment, and a direct comparison with protein A. The platform enabled IgG detection down to 10 ng mL^−1^, showed negligible cross‐reactivity toward IgA and albumin, and outperformed protein A for the two nanoMIPs targeting the Fc region. Overall, this study presents a promising semi‐synthetic strategy for the development of miniaturised, more stable, and potentially multiplexed diagnostic sensors.

## 2 Results and Discussion

### 2.1 Design of IgG Peptides to Imprint via Epitope Discovery

**FIGURE 4:**
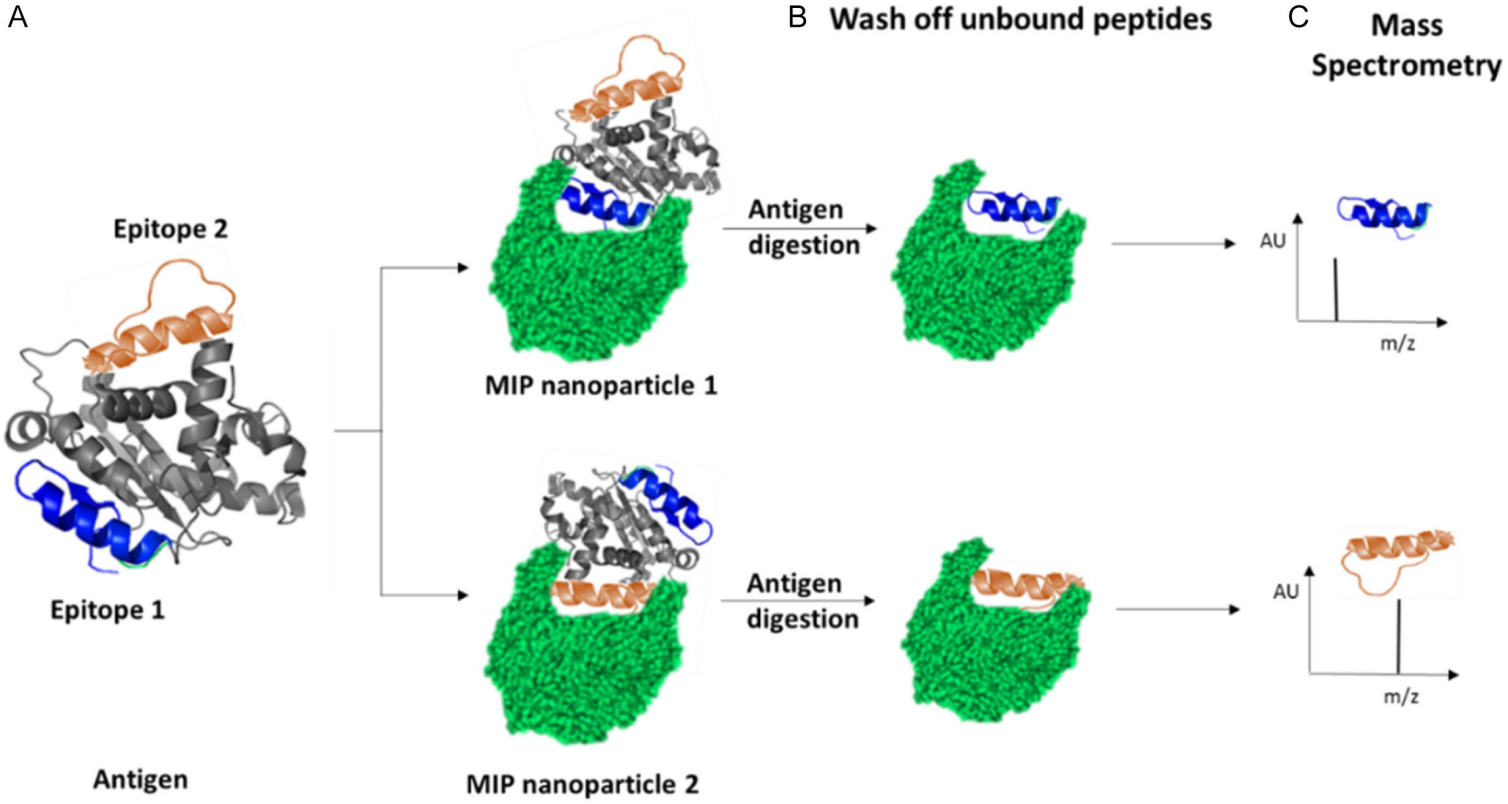
Schematic description of the epitope discovery process. NanoMIPs are polymerised in the presence of the whole protein (A); after enzymatic digestion, unbound peptides and unreacted monomers removed (B); then, the MIP‐protected peptides are eluted from the nanoMIPs and mass spectrometry run on these peptides to identify the aminoacidic sequence (C). Image reproduced with permission from [53].

As previously discussed, with the aim of orienting the binding to IgG, identifying which portion of the antibody to imprint will have an impact on the final performance of the detection system. Understanding which peptide will allow a better binding of the IgG to nanoMIPs is similarly of crucial importance. Over the years, many bioinformatic programs have been developed to predict the potential antigenicity of peptides [48] or simulate protein–ligand interactions, with docking programs like Molegro (http://molexus.io/molegro‐virtual‐docker/) and AutoDock (https://autodock.scripps.edu/). However, a similar approach with nanoMIPs is still in development [49, 50]. A strategy for empirical identification of epitopes (epitope discovery) with potential antigenic properties using molecular imprinting has recently been developed by Piletsky et al. [51] and employed in our protocol. The data from this empirical approach were compared to the known crystallised structures of IgG from the Protein Data Bank (PDB, https://www.rcsb.org/) and used to guide the selection of epitopes to imprint. Antibodies can be divided into distinct classes and subclasses based on differences in the structure of their heavy chains (*α*, *δ*, *ε*, *γ*, *μ*) resulting respectively in IgA, IgD, IgE, IgG, and IgM. Among the five different classes of antibodies, IgGs are the most abundant (13.5 mg mL^−1^) and the second longest‐lived protein circulating in the bloodstream [23, 52]. The epitope discovery methodology consists of the polymerisation of nanoMIPs in the presence of the whole protein of interest (Figure 4A). The complex protein‐nanoMIPs is then exposed to enzymatic digestion (Figure 4B), followed by removal of unbound peptides (Figure 4C) and analysis of the undigested peptides via mass spectrometry. The experimental procedure is based on the assumption that the undigested peptides are protected by the binding pocket of the nanoMIPs, polymerised around it, and therefore protected from digestion. Then, the MIP‐protected peptides are eluted from the nanoMIPs and mass spectrometry run on these peptides to identify the amino acidic sequence. The most abundant peptides detected by mass spectrometry analysis are likely to be the epitopes most exposed on the surface of the protein during the imprinting step, and therefore likely to be immunogenic [54].

As Piletsky et al. have recently demonstrated, when epitope discovery was applied to acetylcholinesterase (AChE), four out of seven peptides identified via the MIP‐based Epitope Discovery approach (ED) matched immunogenic epitopes known in the literature [53]. Epitope Discovery was therefore applied to identify suitable peptides that could be used for the development of nanoMIPs, aimed to bind different regions of IgG. Commercially available IgG was used as a model antibody.

#### 2.1.1 PyMOL and Alignment Analysis

Raw data from the mass spectrometry were processed using ProteinLynx Global SERVER (Waters Corporation, Milford, Massachusetts, USA), and ProteinLynx Global SERVER was used to assemble the data for peak picking, peptide and protein identification, and limited upstream statistics. The most abundant peptides resulting from the analysis performed were likely to be the “most imprinted” and therefore exposed on the surface of IgG. Two of them were from the constant region of the heavy chain of IgG (Ig *γ*_1_ constant region), with amino acid sequences FNWYVDGVEVHNA and EPQVYTLPPSR. A summary of the peptides’ respective positions is reported in Table S1.

PyMOL was used to visualise the positioning of the peptides on the crystal structure of IgG. The crystal structure of human IgG from the Protein Data Bank (PDB) database was used as a model (1HZH, Figure S1) [55].

Peptide 3_FNW (orange section) and 4_EPQ (red section) are located in the constant region of *γ* heavy chain. To visualise the positioning of the peptides on the crystal structure of IgG relative to the binding of protA, the crystallised structure of protA complexed with the constant region of IgG (5U4Y) was aligned to IgG (1HZH) in PyMOL, and the resulting figure is reported (Figure S2) [57]. ProtA, structurally composed of three alpha helices, is highlighted in purple (Figure S2A), and its polar contacts to IgG displayed in Figure S2B.

**FIGURE 5:**
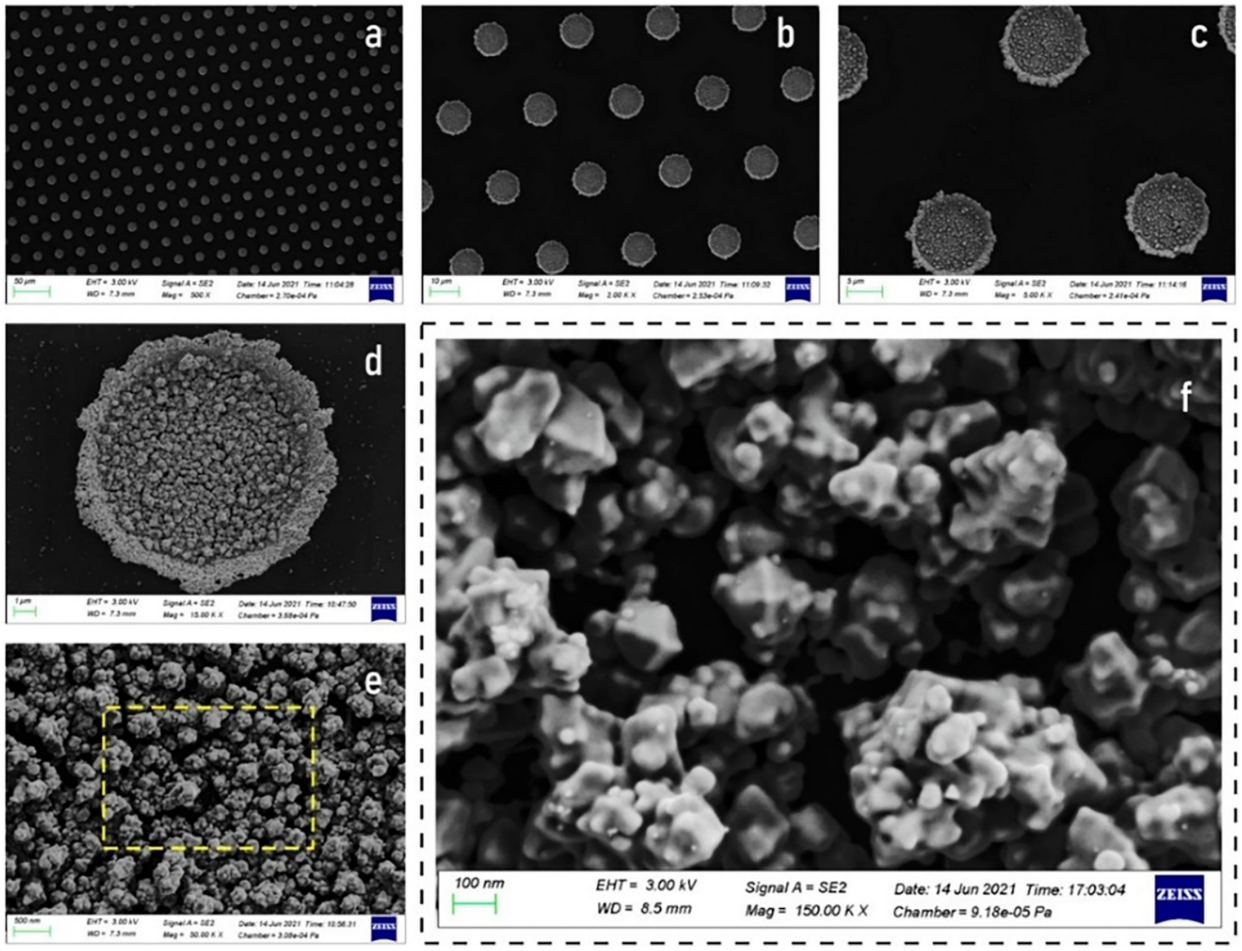
SEM images of gold nanoparticle clusters taken at low magnification (a–c); SEM micrographs of the samples taken at higher magnification (d–f) illustrating the particle morphology at the nanoscale.

Both peptides 3_FNW and 4_EPQ located in the Fc region of the antibody (3_FNW and 4_EPQ), are spatially close to the protA‐binding region and have the potential of being a favourable region for the binding to nanoMIPs. Both peptides were also screened against the library of all known proteins in H*omo sapiens* using the blast tool form the NCBI (https://blast.ncbi.nlm.nih.gov/), and no relevant similarities were found. The data resulting from the epitope mapping, alignment and visual representation of the peptides positioning drove the decision of using the four peptides mentioned for the generation of nanoMIPs.

### 2.2 Characterisation of the Nanostructured Device

The silicon plasmonic surfaces were fabricated and characterised by the BIO and Nano Engineering and Technology for Medicine group (BioNEM) based at the University Magna Graecia (UMG) of Catanzaro. The design and synthesis of nanoMIPs imprinted for IgG was performed at the University of Leicester, with intellectual contribution from Tozaro Ltd.

### 2.3 Characterisation of the Plasmonic Surface Patterned With Gold Nanoclusters

#### 2.3.1 Characterisation via Scanning Electron Microscopy (SEM)

To assess the morphology of the clusters, SEM images of the samples were taken. SEM images with magnification factor from 500 to 5000× (Figure 5a–c) illustrate that the clusters of gold nanoparticles extend over areas of several hundred micrometers with no interstitial or vacancy defects in the patterns. They show a predominantly regular distribution with an average diameter of approximately 10 µm, arranged in a hexagonal lattice with a centre‐to‐centre spacing of 20 µm

SEM images of the samples taken at higher magnification (Figure 5d,e) show that, in each cluster, gold nanoparticles are densely packed, exhibiting an imperfect rhombohedral shape and a size that ranges from about 50 nm to nearly 200 nm for the larger structures (Figure 5f). The extent of this scale interval suggests that particles in the cluster may have a dendritic structure, with smaller particles more numerous than larger particles and the same geometrical motifs occurring periodically at different length scales. Atomic force microscopy (AFM) images of the gold nanoclusters were also performed for the characterisation of the surface (results included in the Supporting information file).

#### 2.3.2 Characterisation of the Functionalised Plasmonic Gold Nanoparticles via Raman Spectroscopy

To verify the successful immobilisation of protA and binding of anti‐tubulin Ab to gold nanoclusters, Raman spectroscopy was applied (Figure S10). Raman maps were collected and analysed, revealing intensity at points 1178 and 1434 cm^−1^, corresponding to histidine and tyrosine signals, respectively, and the CH_2_ bending peak [57]. Figure S10C shows the distribution of Raman intensity for the 1178 cm^−1^ peak; the same results were obtained for the 1434 cm^−1^ point, evidencing a homogeneous distribution of the anti‐tubulin Ab on the gold nanoclusters (AuNPs), while no signal was recorded in the surrounding area.

Raman spectra were also recorded at each step of the functionalisation of the substrate. The spectra evidence an initial increment of the typical proteins’ signals between gold MUDA and the addition of protA: the peak of amide I around 1630 cm^−1^ is present as a shoulder assigned to the C═O stretching of the carbonyl groups, also presents in MUDA. Nevertheless, the peak at ~1335 cm^−1^, attributable to NH bending and C─N stretching mode of amide III, is proportionally increased respect to the total spectrum after protein A functionalisation as well as the at ~1170 cm^−1^ backbone skeletal stretch signal [58]. The antibody–protein interaction does not show a significant difference in Raman fingerprint. What is observable, instead, is a global decrease of Raman intensity, due to the incremented distance between the added molecules and the SERS substrate [59].

Moreover, tryptophan, peak at ~1550 cm^−1^, is one of the amino acids most influenced by protein aggregation and conformational changes from external stimuli. The binding between protA and the antibody is precisely highlighted by the increment of the 1550 cm^−1^ peak, recognising changes in both secondary and tertiary structure of the antibody [58]. When the interaction with the anti‐tubulin Ab occurs, the total signal decreases and only the 1434 cm^−1^ (CH2 bending) peak, in addition to histidine and tyrosine signals at 1178 cm^−1^ become more prominent [57].

#### 2.3.3 Evaluation the Immobilisation of NanoMIPs on Gold Nanoclusters

**FIGURE 6:**
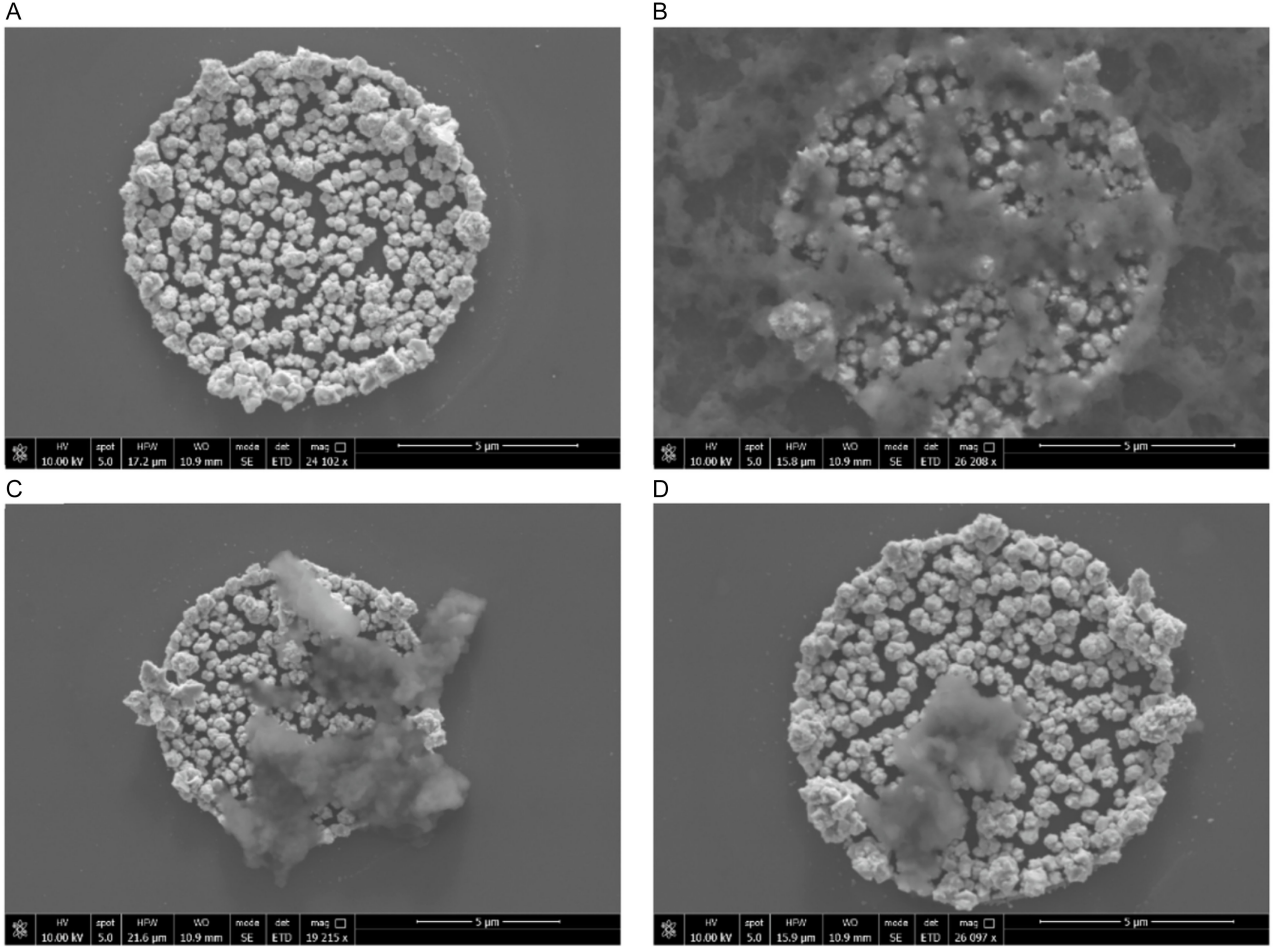
SEM image of gold cluster (*d* = 10 μm), before functionalisation (A); with nanoMIPs (1_VDN_coreshell) dried on the surface (B); and after nanoMIPs immobilisation (C,D); scale bar 5 μm.

Before attempting a sandwich format, the immobilisation of nanoMIPs on gold nanoclusters was confirmed by SEM images. Due to nanoMIPs’ low conductivity, without a conductive metal coating, characterising nanoparticle morphology with sufficient resolution remains challenging and obtain a resolution capable of distinguishing individual particles. However, in this case, if nanoMIPs would have been coated with gold or platinum, it would have been remarkably challenging to differentiate them from the gold nanoparticles forming the clusters, which are similar in size. For this reason, for the purpose of comparison, images of the gold clusters without functionalisation (Figure 6A) and with nanoMIPs dried on the clusters surface (Figure 6B) were taken in parallel to images of a washed surface after EDC/NHS coupling of nanoMIPs (Figure 6C,D).

As observable from the SEM images, bare gold clusters (Figure 6A) appear formed of well‐defined gold nanoparticles, compared to Figure 6B, where a clear layer of opaque‐looking polymer is covering the surface. In Figure 6C,D, nanoMIPs are localised on the gold clusters, with the appearance of a cluster of nanoMIPs rather than individual nanoparticles.

### 2.4 Anti‐IgG DyLight 488 Concentration Optimisation

After nanoMIP immobilisation was confirmed (SM paragraph 1.6 “Characterisation of Peptide‐Imprinted nanoMIPs”), the following step was to identify the optimal concentration of fluorescent anti‐IgG (IgG_D488). This concentration should ideally be the minimal amount possible while still being visible to avoid saturating the signal. Dilution series of IgG_D488 [0.001–1000 μg mL^−1^] were drop‐casted on the platform and the fluorescence recorded (Figure S3A,B).

The concentration of IgG_D488 most suitable for the detection was found to be between 1 and 10 μg mL^−1^. In the following experiment, two concentrations of IgG_D488 were further investigated. In this case, IgGs in a concentration range between 0.01 and 100 μg mL^−1^ were covalently immobilised via EDC/NHS coupling on the gold clusters. After removing the excess of non‐immobilised IgG, IgG_D488 at respectively 1 and 10 μg mL^−1^ were added. Following further washes to remove the excess of IgG_488, the fluorescence was recorded (Figure S3C,D). A sensor platform in the absence of IgG was used as negative control.

The fluorescence recorded was transformed into relative fluorescence units (RFU) applying the analysis described in the methods (*Microscope analysis method*), and the values were plotted accordingly (Figure S4). A concentration dependent response is still observable when using 1 μg mL^−1^ of IgG_D488; however, the sensitivity of the detection significantly improves when 10 μg mL^−1^ is used, with a calculated Z’ of 0.85. This concentration was used in the following experiments.

#### 2.4.1 Evaluating the Binding of NanoMIPs to IgG

Core‐shell nanoMIPs imprinted for the four epitopes were immobilised on the sensing platform. Different concentrations of IgG were tested using a fixed concentration of IgG_D488 (Figure S5A‍–‍D). The graphs reported in Figure S5C,D show a concentration‐dependent response for all the four nanoMIPs tested, whereas negligible fluorescence was found in the control where nanoMIPs were immobilised and IgG_D488 added, likely originating from non‐specific binding (NSB) of IgG_D488 to nanoMIPs. The lowest concentration of IgG recorded was between 0.1 and 0.01 μg mL^−1^.

### 2.5 Testing the Binding in Complex Mixtures

The experiments performed so far were conducted in ideal conditions, where nanoMIPs, IgG, and IgG_D488 were diluted in buffer and no external components were interfering with the interaction between the three binding elements. However, an ideal detection system should perform in complex mixtures, where the least sample treatment (e.g. purification, filtering) is applied. To verify the robustness of the detection system developed, a mixture of proteins and other components was used during binding tests using 1_VDN_coreshell and 3_FNW_coreshell nanoMIPs (Figure S6).

In the absence of purified human plasma with quantified levels of IgG, a solution containing plasmatic proteins (20%), of which at least >95% is human albumin and excipients (sodium chloride, sodium caprilate, acetyltriptophane, sodium 123.5–136.5 mmol L^−1^) was spiked with IgG concentrations and used instead.

A drop in detection is, however, expected and well‐documented often due to non‐specific binding to the pool of proteins present in plasma and serum [60]. IgG concentrations in human plasma typically range between 6.0 and 16.0 mg mL^−1^. The detection window in the developed system is between 1 × 10^−5^ and 1 mg mL^−1^; the matrix can, therefore, be diluted up to 600,000 times, and the target can still be detectable. Assay companies are nowadays including in their detection systems an internal standard made of already diluted plasma solutions, in order to match the values found in real diluted samples [60]. A drop in detection in these conditions is therefore manageable. Recently, Ventisette et al. demonstrated the successful application of a molecularly imprinted polymer‐based sensing platform for the selective detection of human IgG1 directly in human serum, also demonstrating selectivity against other immunoglobulin classes and across the four IgG isotypes. Their study provides an important benchmark for the clinical applicability of MIP‐based IgG sensors [61]. In contrast, the present work focuses on the development of a fundamentally different sensing architecture, based on Fc‐directed epitope‐imprinted nanoMIPs integrated onto a plasmonic MEF substrate for oriented IgG recognition. While the synthetic plasma‐like matrix used in this study enabled a controlled evaluation of matrix effects, validation using authentic clinical serum samples will represent the next step toward the translation of the proposed platform.

### 2.6 Cross‐Reactivity Tests

#### 2.6.1 Cross‐Reactivity Tests Using Equivalent Protein

The following step in the development of the detection system was to test the binding specificity of the nanoMIPs towards the portion of IgG they were imprinted for, as well as cross‐reactivity towards different proteins. In order to do so, all four nanoMIPs were tested against albumin, the most abundant protein in human plasma (35–50 mg mL^−1^ in healthy subjects) [62].

For experimental convenience, its homologue BSA was used instead of human albumin, at the same concentrations of IgG tested and compared to the sensor platform with IgG (Figure S7).

From the experiment reported in Figure S7, the signal registered when BSA was used in place of IgG is as low as the control in the absence of IgG, showing no cross selectivity with IgG.

#### 2.6.2 Specificity and Selectivity Assessment of IgG‐Imprinted NanoMIPs

To further assess the specificity of the nanoMIP‐based platform, additional controls were performed beyond the BSA cross‐reactivity test. First, all four IgG‐imprinted nanoMIPs were tested against BSA, used as an albumin model, over the same concentration range used for IgG. In all cases, the fluorescence signal obtained with BSA was comparable to the control in the absence of IgG, indicating negligible cross‐reactivity toward albumin under the tested conditions (Figure S11).

IgG‐imprinted nanoMIPs were then compared with control‐MIPs possessing a similar polymeric structure but imprinted for a different template. While the IgG‐imprinted nanoMIPs produced fluorescence patterns localised on the gold clusters, the control‐MIPs generated a more diffuse signal with no clear correlation with the plasmonic structures, supporting the template‐dependent nature of the binding response (Figure S12).

Selectivity was also evaluated across different antibody classes and light‐chain variants. Prior to these experiments, sequence alignment analysis was performed by comparing each selected peptide epitope against human proteins and different immunoglobulin classes (Table S4). The Fc‐directed nanoMIPs, 3_FNW_coreshell and 4_EPQ_coreshell, were tested against IgA, which does not contain the selected γ‐heavy‐chain Fc epitopes. Both nanoMIPs showed a markedly lower response toward IgA than toward IgG, indicating preferential recognition of IgG‐related Fc regions (Figure S13). In contrast, the light‐chain‐directed nanoMIPs showed higher cross‐selectivity between IgGκ and IgGλ variants, suggesting that these candidates were less suitable for selective IgG orientation (Figures S14 and S15). Based on these results, the Fc‐imprinted nanoMIPs were selected as the most promising candidates for protein A‐like IgG recognition on the plasmonic platform.

#### 2.6.3 Selectivity Tests Across Different Classes of Antibodies

The last experiment performed was testing the nanoMIP selectivity against antibodies that do not have the template peptides in their sequence. IgG shares *κ* and *λ* light chains with the remaining four classes of antibodies (IgA, IgE, IgD, IgM), while the constant region of the heavy chain (*γ*) is unique to each class. However, Ig domains are heavily conserved and some portions can also be found in different proteins. Before the experiment was performed, alignment was run using protein blast form the NCBI (https://blast.ncbi.nlm.nih.gov/) between each of the peptides and the whole pool of human proteins as well as each peptide against each class of Ig. A summary of the results from the alignment is reported in Table S2.

When 3_FNW_coreshell nanoMIPs were tested against IgA (Figure S8), they showed an effective discrimination between IgG and IgA with 9.8 % of cross‐selectivity to IgA at the highest concentration tested (100 μg mL^−1^) and below the control for the remaining concentrations tested. Similar results were attained with 4_EPQ_coreshell nanoMIPs, where the percentage of cross‐reactivity was 15.4% and 11.8%, respectively for 100 and 100 μg mL^−1^.

#### 2.6.4 Comparison With Protein A

A side‐by‐side comparison between nanoMIPs and protA was made by immobilising alternatively 3_FNW_coreshell nanoMIPs, 4_EPQ nanoMIPs, and protA, as shown in Figure S9.

Both nanoMIPs tested perform with a similar trend to protA; however, in both cases, nanoMIPs show a higher percentage of binding than protA, showing the potential to be considered a suitable replacement to their natural counterpart. The difference in percentage between 3_FNW and protA is on an average 20.6% with a minimum of 7.15 and a maximum of 27.2%. 4_EPQ to protA ratio results on an average of 17.8% (10.2% min and 24.04% max).

## 3 Conclusion

The strategy of designing IgG‐specific peptides for molecular imprinting represents a significant advancement in the development of synthetic affinity materials. One of the most compelling strengths of this work lies in the integration of empirical epitope discovery with computational tools such as PyMOL and sequence alignment. This dual approach allowed the selection of structurally exposed and immunologically relevant peptides, increasing the likelihood of generating high‐affinity nanoMIPs.

After initial assessment of the integration of protein A with the nanofabricated platform was performed, Ig‐imprinted nanoMIPs were, for the first time, successfully integrated with a nanofabricated plasmonic platform with gold nanoclusters.

The epitopes for imprinting were carefully selected via a MIP‐based epitope discovery approach and a sandwich array successfully developed using nanoMIPs imprinted for the Fc region of IgG, with a detection limit of 10 ng mL^−1^. The signal was generated by fluorescent anti‐IgG antibodies and the detection enhanced via the metal enhanced fluorescence (MEF) phenomenon. NanoMIPs demonstrated to be specific to the IgG class with no cross selectivity to IgA. NanoMIPs as synthetic protein A were tested in parallel to protein A, outperforming it on an average by 20.6% and 17.8% for the two Fc‐nanoMIPs imprinted.

From a materials science perspective, the successful patterning of gold nanoparticles on silicon, combined with SEM and AFM characterisation, ensures a well‐defined and reproducible nanostructured substrate.

Fluorescence optimisation also yielded highly promising results. The use of DyLight 488–conjugated anti‐IgG enabled a concentration‐dependent response, with the best signal obtained at 10 µg mL^−1^. A Z’ factor of 0.85 confirms the excellent reliability of the detection platform.

Another noteworthy achievement is the ability of the system to maintain functionality even in complex protein mixtures, simulating plasma conditions. Although a drop in detection was observed, as expected, the detection range (from 10 to 1 mg mL^−1^) remains within physiologically relevant concentrations. Furthermore, selectivity was rigorously confirmed, with low cross‐reactivity toward IgA and albumin, indicating that the nanoMIPs selectively recognise IgG even in challenging environments.

The sensor platform demonstrated the potential for the automation in an automatic reader and can be further developed for the fabrication of semi‐synthetic and miniaturised arrays. Due to the versatility and adaptability of nanoMIPs and the micro scale of the platform, the developed detection system has the potential of becoming a universal and multiplexed platform for the detection of proteins or/and oncometabolites for which antibodies detection is unable.

### 3.1 Limits

Although the results obtained in this study are promising and demonstrate the feasibility of using epitope‐imprinted nanoMIPs for the selective detection of IgG, it is necessary to acknowledge certain limitations. Mass spectrometry may favour more stable or more easily ionisable peptides, introducing a potential bias in the identification of epitopes [63].

Secondly, although structural analysis with PyMOL and sequence alignment provided valuable insights, the selection of only four epitopes may have limited the diversity of binding sites explored. Broader coverage could improve the specificity of the system. From an experimental standpoint, the tests were conducted under ideal buffer conditions. Although some tests were performed in complex mixtures, a thorough validation in real matrices is still lacking.

### 3.2 Future Prospective

To transform this platform into a system truly applicable in clinical or diagnostic settings, several advancements will be required. It will be essential to test the system on real biological samples, such as plasma or serum from healthy individuals and patients, in order to assess the robustness of the binding and the sensor’s ability to operate in the presence of potential interferences. Consequently, it will be important to extend the device validation to different IgG subtypes. Furthermore, it will be necessary to determine whether the sensor can be reused multiple times or should be employed as a single‐use device. Finally, a cost–benefit analysis will be required, starting from the production of the nanoMIPs to the large‐scale manufacturing of the devices.

## 4 Materials and Methods

### 4.1 Experimental Design

**FIGURE 7:**
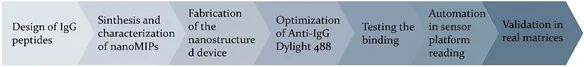
Workflow. *Design of IgG peptides*: Identification of specific IgG epitopes to serve as templates for molecular imprinting; *Synthesis and characterisation of nanoMIPs*: Fabrication of molecularly imprinted polymeric nanoparticles and verification of their physicochemical properties; *Fabrication of the nanostructured device*: Integration of nanoMIPs onto a solid substrate to build the sensing platform; *Optimisation of anti‐IgG DyLight 488 concentration*: Fine‐tuning of the fluorescent probe concentration to maximise specific signal output; *Testing the Binding*: Evaluation of the sensor’s selectivity and sensitivity in realistic or biologically complex samples; *Automation in sensor platform reading:* Implementation of automated systems for rapid, consistent signal acquisition and data analysis.

The main objective of this study is to develop and validate an innovative sensor based on molecularly imprinted polymeric nanoparticles (nanoMIPs) specifically designed to selectively and directionally recognise immunoglobulin G (IgG). Figure 7 shows the workflow of the experimental process.

### 4.2 Fabrication of the Nanostructured Device

#### 4.2.1 Optical Lithography on Silicon Wafers

**FIGURE 8:**
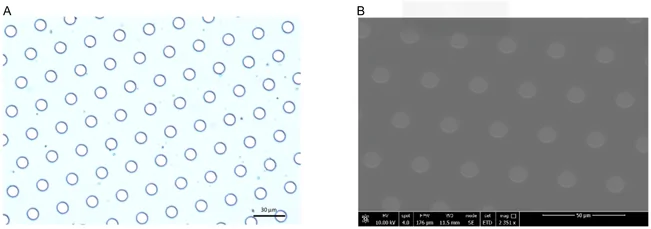
Lithographed silicon wafer, optical microscope image, scale bar 30 µm (A); SEM image, scale bar 50 µm (B).

In order to obtain ordinated and reproducible patterns, optical lithography was utilised with a protocol adapted from Battista et ‍al. [64]. Silicon wafers P type (100) with a resistivity of 10 Ω/cm were used as a substrate for the subsequent deposition of gold clusters. Positive photo‐resist (S1813) was deposited on the wafer, and optical lithography was applied to transfer a pattern of micro‐holes with a diameter of *d* = 10 µm and a centre‐centre distance of 30 µm (Figure 8). When the resist was developed, gold clusters were deposited via electroless deposition as described in the following section.

### 4.3 Optimisation of Electroless Deposition of Gold Clusters

Electroless deposition is an autocatalytic chemical reduction of metal ions in an aqueous solution in the presence of silicon nanoparticles. Gold ions exchange electrons with the silicon substrate that is the reducing agents itself. The reaction occurs in a 0.15 M solution of hydrofluoric acid (HF) containing the silicon substrate and auric chloride (AuCl_3_). The driving force in this process is the difference between the redox potentials of the two half‐reactions, gold reduction and silicon oxidation, which depends on solution temperature, concentration and pH, explaining why these parameters also influence the particles size and density [64]. For this configuration, there are the following reactions, at the anode (that is, silicon oxidation)

(1) $$\mathrm{Si}+2H_{2}O\to {\mathrm{SiO}}_{2}+4H^{+}+4e^{-}$$

And at the cathode (that is, gold reduction)

(2) $${\mathrm{Au}}^{3+}+3e^{-}\to {\mathrm{Au}}^{0}$$

**FIGURE 9:**
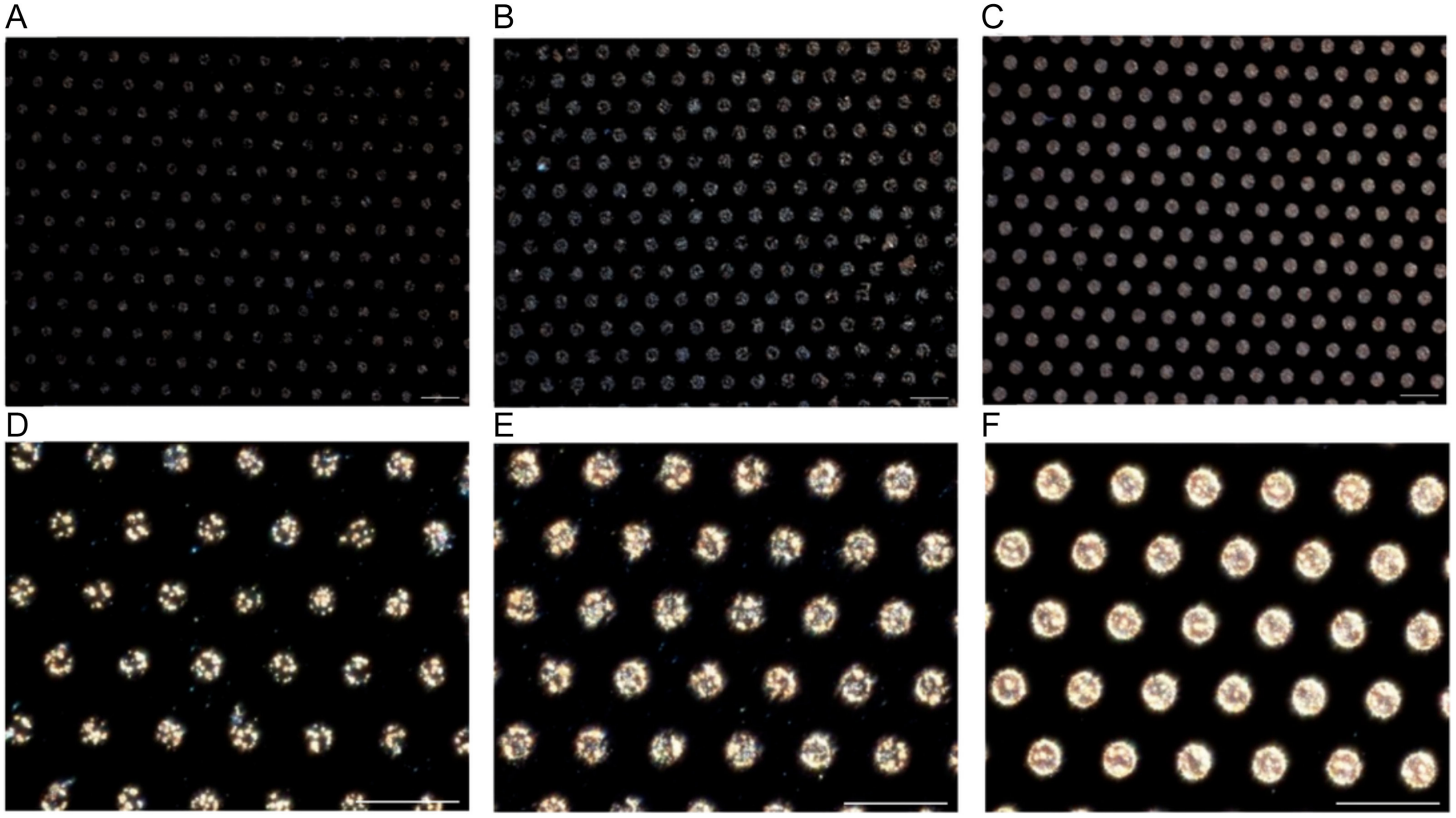
Gold deposition on silicon substrates; 60 s (A), 120 s (B), 180 s (C); 50× magnification 60 s (D), 120 s (E), 180 s (F); scale bar 30 µm.

In order to achieve an optimal gold clusters’ growth, a solution of auric chloride (5 mM) was used, and the effect of variation in temperature (30, 45 and 55 °C) and immersion times (60, 120 and 180 s) was investigated. Among all temperatures tested, 45 °C was found to be the optimal. At lower temperatures, the gold clusters still form; however, the growth is slower and less uniform across the surface. At temperatures above 50 °C, the resist layer was damaged, resulting in overgrowth under the layer, with uncontrolled shape. For this reason, the temperature was set to 45 °C, and the immersion time varied (Figure 9).

As shown in Figure 9, prolonging the immersion time resulted in increase in gold content in each cluster. Above 4 min, the solution started to be deposited outside of the lithographed holes, an indication of saturation of the available surface inside the cavity. For this reason, 180 min and 45 °C were chosen for the gold deposition.

### 4.4 Integration of NanoMIPs With Sensor Platform for the Detection of IgG

**FIGURE 10:**
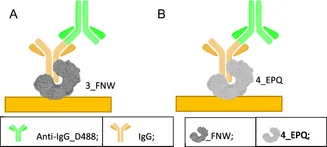
Schematic representation of nanoMIPs immobilised on gold clusters binding IgG, fluorescent IgG (IgG_D488) in sandwich format. Fc binding nanoMIPs 3_FNW (A), Fc binding nanoMIPs 4_EPQ (B) binding IgG and IgG_D488.

As previously discussed, nanoMIPs were synthesised using the core–shell approach (3_FNW_coreshell, 4_EPQ_coreshell). NanoMIPs were immobilised on the gold clusters via EDC/NHS coupling between the NH_2‐_ groups of the nanoMIP amino‐shell and the carboxyl groups of the functionalised gold clusters. Four nanoMIPs were functionalised: 3_FNW_coreshell and 4_EPQ_coreshell (Figure 10A) for binding to the Fc region of the antibody (protA‐like nanoMIPs), 1_VDN_coreshell (Figure 10B) for binding to the κ light chain, 2_YAA_coreshell directed to the *λ* light chain.

To detect the fluorescent signal, a fluorescently labelled anti‐IgG able to bind both heavy and light chains (Goat anti‐Human IgG (H&L), DyLight 488 conjugated, Agrisera) was used in a sandwich format.

### 4.5 Microscope Analysis Method

Images acquired were analysed using the NIS‐Elements 4.30 software provided by Nikon Instruments Inc. Images were thresholded in TRITC using 950 ≤ intensity values ≤ 2050 ± 50, smooth 12×, clean 12×, fills holes and separation 4×, 10.4 ≤ size (μm) ≤20.4 and circularity between 0 and 1. Threshold was applied to images in each channel. A minimum of 600 events per image and 5 a minimum of images was analysed each sample, and the average was taken. Baseline was taken each time on the negative control.

## Author Contributions

**Natalia Malara** **,** **Fabiana Grillo** **,** **Francesco Gentile** **:** conceptualisation. **Maria Laura Coluccio** **,** **Nilde Fera** **,** **Virginia Garo** **,** **Elena Piletska** **,** **Donald Jones** **,** **Francesco Canfarotta** **:** methodology. **Fabiana Grillo** **:** investigation. **Francesco Canfarotta** **,** **Elena Piletska** **,** **Donald Jones** **,** **Thong H. Cao** **:** validation. **Maria Laura Coluccio** **,** **Sergey Piletsky** **,** **Francesco Gentile** **,** **Patrizio Caldeloro** **:** visualisation. **Natalia Malara** **,** **Sergey Piletsky** **,** **Francesco Gentile** **,** **Patrizio Caldeloro** **:** supervision. **Fabiana Grillo** **,** **Sandro Dragone** **:** writing – original draft. **Fabiana Grillo** **,** **Natalia Malara** **,** **Francesco Gentile** **,** **Maria Laura Coluccio** **:** writing – review and editing.

## Funding

The authors have nothing to report.

## Conflicts of Interest

The authors declare the following competing financial and non‐financial interests: Francesco Canfarotta is the Head of Tozaro Ltd. and provided a scientific contribution to the synthesis of the molecularly imprinted polymers (MIPs) used in this study. However, Tozaro Ltd. provided no financial or economic support, direct or indirect, for the execution of this research. The authors received no compensation, reimbursement, or financial benefits from Tozaro Ltd. at any stage of the work. Accordingly, the authors declare no other competing interests. All authors declare they have no competing interests.

## Supporting information

Supplementary Material

## Data Availability

Data and materials are available upon request by contacting the corresponding author via email.
