# Supplementary material for: Integration of NanoMIPs With Gold‐Patterned Plasmonic Surfaces as Synthetic Protein A
Source: Small Sci. 2026 Sep 2;6(9):e70394. doi: 10.1002/smsc.70394 (PMC13574059; doi:10.1002/smsc.70394)
Supplement: Supplementary file 1 [file SMSC-6-e70394-s001.pdf]

Supporting Information

Integration of nanoMIPs with gold-patterned plasmonic surfaces as synthetic protein A

Fabiana Grillo, Francesco Gentile, Francesco Canfarotta, Maria Laura Coluccio, Elena Piletska, Donald Jones, Thong H. Cao, Virginia Garo, Nilde Fera, Sandro Dragone, Patrizio Caldeloro, Sergey Piletsky, Natalia Malara**

This PDF file includes:

Supplementary Text
Figs. S1 to S23
Tables S1 to S6
References (1 to 5)

Supplementary Text - List of abbreviations

Ab	Antibody
AChE	Acetylcholinesterase
AEAPTMS	N-[3-(Trimethoxysilyl)propyl]ethylenediamine
AFM	Atomic force microscopy
APTES	(3-Aminopropyl)triethoxysilane
AuNPs	Gold nanoparticles
BSA	Bovine serum albumin
DLS	Dynamic light scattering
EDC	(3-Dimethylaminopropyl)-N′-ethylcarbodiimide
ELISA	Enzyme linked immunosorbent assay
Fc	Fragment crystallisable
FITC	Fluorescein isothiocyanate
HF	Hydrofluoric acid
IO-NPs	Iron oxide nanoparticles
Ig	Immunoglobulin
IgA	Immunoglobulin A
IgD	Immunoglobulin D
IgE	Immunoglobulin E
IgG	Immunoglobulin G
IgG_D488	Fluorescent anti-IgG Dylight 488
IgM	Immunoglobulin M
IVD	In vitro diagnostics
LoQ	Limit of quantification
MEF	Metal enhanced fluorescence
MIP	Molecularly imprinted polymer
MUDA	11-mercaptoundecanoic acid
NanoMIPs	Molecularly imprinted polymer nanoparticles
NaOH	Sodium hydroxide
NPS	New psychoactive substances
NSB	Non-specific binding
PBS	Phosphate buffered saline
PDB	Protein Data Bank
Pep1_VDN	Peptide 1 VDN (sequence)
Pep2_YAA	Peptide 2 YAA (sequence)
Pep3_FNW	Peptide 3 FNW (sequence)
Pep4_EPQ	Peptide 4 EPQ (sequence)
ProtA	Protein A
SEM	Scanning electron microscopy
SERS	Surface-Enhanced Raman Spectroscopy
S/N	Signal to noise
SPR	Surface plasmon resonance
TRITC	Tetramethylrhodamine
UMG	University Magna Graecia
USD	US dollars
UV	Ultraviolet
Z’	Zeta prime

1. Synthesis and characterization of nanoMIPs imprinted for IgG

1.1. Glass Beads Activation

A total of 300 g of glass beads (70–110 µm) were thoroughly cleaned using three volumes of water and one volume of ethanol under vacuum. They were then boiled in 4 M NaOH (300 mL) for 15 minutes, followed by extensive rinsing with water (3 × 2 L). The beads were subsequently treated with 50% sulfuric acid (120 mL) for 1 hour and again washed with water (2 × 2 L). They were then incubated for 5 minutes in phosphate buffer (PBS 0.01 M or phosphate buffer 0.1 M, 200 mL). To ensure a final pH between 6 and 8, ten additional water washes (10 × 2 L) were performed. The solid phase was dried with acetonitrile (2 × GB volume) under vacuum for 15 minutes and finally placed in an oven for 2 hours at 150 °C.

1.2. Glass Beads Silanisation

Three silanes (see Table S3) were individually added to a toluene solution (0.4 mL per gram of GBs) at 80 °C, along with BTSE (initially 0.12% v/v, later increased to 2.4% v/v). A nitrogen-flushed Duran bottle was used to mix the warm glass beads and toluene-silane solution. The mixture was sealed and swirled to suspend the beads, then left to react overnight at 80 °C. After the reaction, the beads were washed with methanol (3 × GB volume) and acetonitrile (5 × GB volume) to remove residual silanes. They were dried under vacuum and then further dried in an oven at 150 °C for 2 hours. A sample was collected for analysis, and the remaining beads were incubated in DI water overnight at 40 °C. Final washes were performed with DI water (5 × GB volume and 3 × GB volume), followed by drying (2 h, 150 °C) and storage at room temperature. Another sample was collected for analysis.

1.3. Glass Beads Wash Simulation and Amino Groups Quantification

To test silane stability, GBs were subjected to rigorous washing with DI water (10 × GB volume at room temperature) and ethanol (10 × GB volume at 65 °C). Triplicate samples (3 × 1 g) were taken for analysis. A calibration curve was prepared using a 1:1 dilution series of dodecylamine (3 × 1 mL, 0.1–5 mM, 0.93 mg mL^{-1}) in ethanol. The silanised GBs (3 × 1 g) were reacted with 2 mL of ninhydrin (1.07 mg mL^{-1}, 6 mM) in the dark for 180 minutes at 60 °C. After cooling, UV spectra (300–700 nm) were recorded, with a peak at λ_{max} = 580 ± 4 nm, using 6 mM ninhydrin as reference. Based on the optimisation and stability assessment,

glass beads functionalised with 3% v/v AEAPTES and 2.4% v/v BTSE were used for peptide-template immobilisation and subsequent nanoMIP synthesis.

1.4 Template coupling to silanised glass beads

Following activation, silanisation, and stability assessment, the peptide templates were covalently immobilised onto the amino-functionalised glass beads. The coupling strategy was selected according to the functional groups present in each peptide.

1.4.1. Coupling of peptides to silanised-glass beads

The immobilisation of a peptide on glass beads can be achieved via different methods. One of the most common strategies used is covalent immobilisation via the N-terminal of the peptide to the silane on surface of GB using glutaraldehyde (GA).[1] However, when a peptide is composed of amino acids presenting amino groups in their side chain (e.g. lysines (K), arginines (R), hystideines (H)), this type of coupling could lead to a lack of peptide oriented immobilisation. In those circumstances, it is recommended to add a terminal cysteine to the peptide, and immobilise it via the thiol group using *N*-succinimidyl iodoacetate (SIA). Table S5 shows the peptides used in this thesis work, the first two peptides, VDNALQSGNSQ and YAASSYLSLTPEQW (Pep1_VDN, Pep2_YAA), do not have in their sequence any of the amino acids mentioned above (K, R, H) and therefore GA immobilisation was applied.
On the contrary, both Pep3_FNW and Pep4_EPQ have NH2 in the side chain (FNWYVDGVEVHNAK, EPQVYTLPPSR) and an oriented immobilisation would have not been achievable, for this reason cysteine and glycine, were added at the N-terminal and SIA immobilisation was adopted instead.

1.5. Solid-phase synthesis of nanoMIPs: Core-shell synthesis of nanoMIPs in aqueous solvent

To prevent the unfolding of the secondary structure of the peptides, synthesis in an aqueous environment was performed. The protocol used for synthesis in an aqueous environment involved water-soluble acrylate monomers. This is an established protocol,[1] however, in contrast to iniferter-based methods, the non-living character of conventional persulfate-initiated polymerisation gives less control over particle-size distribution and does not allow sequential polymerisation without additional initiators.[2] Imprinting in water also provides environmental advantages, such as reducing organic waste; however, one of the limitations of dithiocarbamate-based iniferters is their poor solubility in water. Hence, a water-soluble

iniferter (WSI), 2-morpholino-2-oxoethyl bis(2-hydroxyethyl)carbamodithioate, was synthesised in-house by MIP Discovery Ltd using a proprietary synthesis and utilised for the preparation of peptide-imprinted core-shell nanoMIPs (Figure S16).
The synthesised iniferter, 2-morpholino-2-oxoethyl bis(2-hydroxyethyl)carbamodithioate, has a calculated LogP of −0.78, compared with 3.81 for N,N-diethyldithiocarbamic acid benzyl ester, due to the more hydrophilic structure of the compound. This WSI was utilised for the core-shell synthesis of nanoMIPs for peptide imprinting. The core was imprinted using a polymeric mixture tailored for binding to peptides, while the amino-shell was made of APMA. The polymerisation mixture was added to the solid support bearing the immobilised template, and polymerisation was initiated via UV irradiation, followed by the removal of unreacted monomers and low-affinity nanoMIPs. Following the first polymerisation step, the amino-shell was grafted by adding APMA to the solid support bearing the template-bound core nanoMIPs, and polymerisation was reactivated. After a second low-affinity washing step, the high-affinity nanoMIPs were collected. APMA is a monomer commonly used in standard aqueous polymerisation mixtures to facilitate binding to peptides and proteins. Excluding APMA from the core would favour its localisation in the shell, effectively protecting the binding site from unwanted reactions that could potentially reduce or invalidate MIP binding to the target. However, in this specific case, the selected peptides Pep1_VDN and Pep4_EPQ possess relatively low isoelectric points, 3.4 and 6.14, respectively, and the presence of APMA in the core was considered beneficial for the interaction between the MIPs and the templates. For this reason, a limited quantity of APMA was added to the core using a 1:7.25 core-to-shell ratio, maintaining a higher APMA content in the shell while retaining a smaller amount in the core of the MIPs.

1.6 Characterisation of Peptide-Imprinted nanoMIPs

1.6.1. NanoMIPs characterisation by DLS and UV analysis

Prior to fluorescence optimisation, the peptide-imprinted nanoMIPs were characterised in terms of size, concentration, morphology, and surface immobilisation. Dynamic light scattering (DLS) analysis showed hydrodynamic diameters of 59.61 ± 0.75 nm, 57.90 ± 5.10 nm, 90.17 ± 1.07 nm, and 83.47 ± 1.41 nm for 1_VDN_coreshell, 2_YAA_coreshell, 3_FNW_coreshell, and 4_EPQ_coreshell nanoMIPs, respectively (Table S6). The corresponding nanoMIP concentrations, determined by UV analysis at 197 ± 5 nm using a calibration curve, were 0.58, 0.59, 0.86, and 0.72 mg mL^{-1}, respectively (Figures S17 and S18). SEM analysis was also performed to assess nanoMIP morphology, showing

predominantly globular particles, with some aggregation observed for aqueous core-shell nanoMIPs, likely associated with the amino-functionalised shell.

1.6.2. Investigating nanoMIPs morphology by SEM

NanoMIPs morphology was also studied using scanning electron microscopy (SEM). However, due to the poor conductivity of the polymer, optimisation was required in the SEM settings. Different surfaces were explored for the deposition of nanoMIPs including carbon stab, glass, glow discharged glass and polylysine glass; metal coating comprising carbon-copper, chromium, gold-palladium, and platinum. Out of all the surfaces tried, glow discharged glass was found to be the most suitable for nanoMIPs deposition. Carbon-copper and chromium were not stable when exposed to high beam voltage. Gold-palladium was found to be a considerable improvement compared to the coating tested before. Platinum was the metal permitting the highest contrast and resolution without compromising the stability of the sample. In the case of peptide-imprinted nanoMIPs, core-shell nanoMIPs were compared with MIPs previously synthesised in aqueous environment using radical polymerisation. When compared, both nanoMIPs appear to have a globular-round shape, however, core-shell nanoMIPs showed a higher degree of aggregation on the slide, which might be due to the positive charge given by the amino-shell (Figure S19).

1.7. Immobilisation of peptide-imprinted nanoMIPs on gold nanoclusters

The immobilisation of nanoMIPs onto the gold-nanoclustered plasmonic surface was evaluated by SEM by comparing bare gold clusters, nanoMIPs dried on the surface, and washed surfaces after EDC/NHS coupling. Bare gold clusters appeared as well-defined gold nanoparticles, whereas nanoMIP-functionalised surfaces showed polymeric material localised on the gold clusters. The functional activity of the immobilised nanoMIPs was further assessed by testing IgG binding using a fixed concentration of DyLight 488-labelled anti-IgG, IgG_D488, at 10 μg mL^{-1}. All four nanoMIPs showed a concentration-dependent fluorescence response toward IgG in the range of 0.01–100 μg mL^{-1}, while negligible fluorescence was observed in the control where nanoMIPs were immobilised and IgG_D488 was added in the absence of IgG. The lowest IgG concentration detected was between 0.1 and 0.01 μg mL^{-1}. These results support both the immobilisation and functional availability of the nanoMIPs on the plasmonic sensing platform (Figure S20).

1.8 Evaluation of metal enhanced fluorescence

To ensure the fluorescent signal was amplified via the MEF effect, a fluorescently labelled antibody with DyLight 488 (IgG_D488) was drop-casted and left to dry on the surface of a silicon wafer functionalised with gold clusters (Figure S21 C, D). For comparison, silicon wafers with no gold clusters functionalisation (Figure S21 A, B) were used.

As can be observed, in both cases the majority of the material is deposited on the edges of the drop as a result of the edge effect, however, where no metal clusters are present on the silicon wafer (Figure 21 A), the fluorescent signal is uniformly distributed throughout the surface. On the contrary, in the presence of gold, the fluorescent signal is amplified in correspondence to the gold clusters, generating an average S/N of 18.92 with an average signal on the clusters of 265.90 ± 64.12 and a background (noise) of 14.05 ± 6.5 with an enhancement percentage of 1892 %. This confirms the data already present in the literature.[3]

1.9 Cross-reactivity with control-MIP

A further test was conducted replacing nanoMIPs imprinted for IgG with control nanoMIPs, i.e. nanoMIPs with similar polymeric composition. Control-MIPs were immobilised on the sensing platform in parallel to nanoMIPs imprinted for VDN (Figure S22).

Whilst nanoMIPs imprinted for VDN show specific patterns, denoting binding to the gold clusters (Figure S22 A), in control-MIPs a spread fluorescence across the spots is observable instead, with no correlation with the gold clusters. Only in the platform where the highest concentration of IgG was used (100 μg/mL) some correspondence is visible, however it is more likely to be from the enhancement effect.

1.10 Characterisation of gold nanoclusters via atomic force microscopy

Atomic Force Microscopy (AFM) was used to analyze the surface of the sample (**F**igure S23). The height profile of the particles ranged mostly between 0 and 60 nm, with a few reaching 80 to 120 nm. The 3D and relief density plots (Figure S23 A-B) provided a clear view of surface topography. To quantify surface complexity, the fractal dimension was calculated using the Q parameter, which relates to height variations across different spatial scales. The result was a fractal dimension of $D_f \sim 2.2$ (Figure S12 C), indicating a relatively smooth surface with some 3D structure contributed by clusters of gold nanoparticles. For reference, a perfectly flat (Euclidean) surface has $D_f = 2.2$, while more complex textures fall between 2 and 3.

2. Iron Oxide Nanoparticles Silanisation

Following an adapted protocol from *Bioconjugate Techniques* and optimised by F. Grillo, a 50 mL solution of 5% water in ethanol (v/v) was prepared and adjusted to pH 4.5–5.5 with acetic acid. Silane coupling agents APTES and AEAPTMS were dissolved in this aqueous solution and reacted for 5 minutes at room temperature. After flushing with nitrogen, IO-NPs were added and sonicated continuously for 4 hours. The particles were washed five times with 5% ethanol in DI water (5 × 250 mL), then dried first with nitrogen and subsequently in an oven at 110 °C for 1 hour. Final drying was done overnight in a vacuum chamber.

3. Iron Oxide Nanoparticles Amino Groups Quantification

For each test, 5 mg of silanised IO-NPs were suspended in 2 mL of MilliQ water and sonicated for 10, 30, and 60 minutes. After removing the supernatant, particles were washed (3 × 2 mL DI water) and resuspended in 1 mL ethanol. Ninhydrin (1 mL, 2.14 mg mL^{-1}, 12 mM) was added and samples incubated for 180 minutes at 60 °C in the dark. A dilution series of dodecylamine (3 × 1 mL, 0.1–5 mM, 0.93 mg mL^{-1}) in ethanol was used for calibration. After incubation, samples were cooled and UV spectra recorded (300–700 nm), with λmax = 580 ± 4 nm. Each test was conducted in triplicate.

Fig. S1.

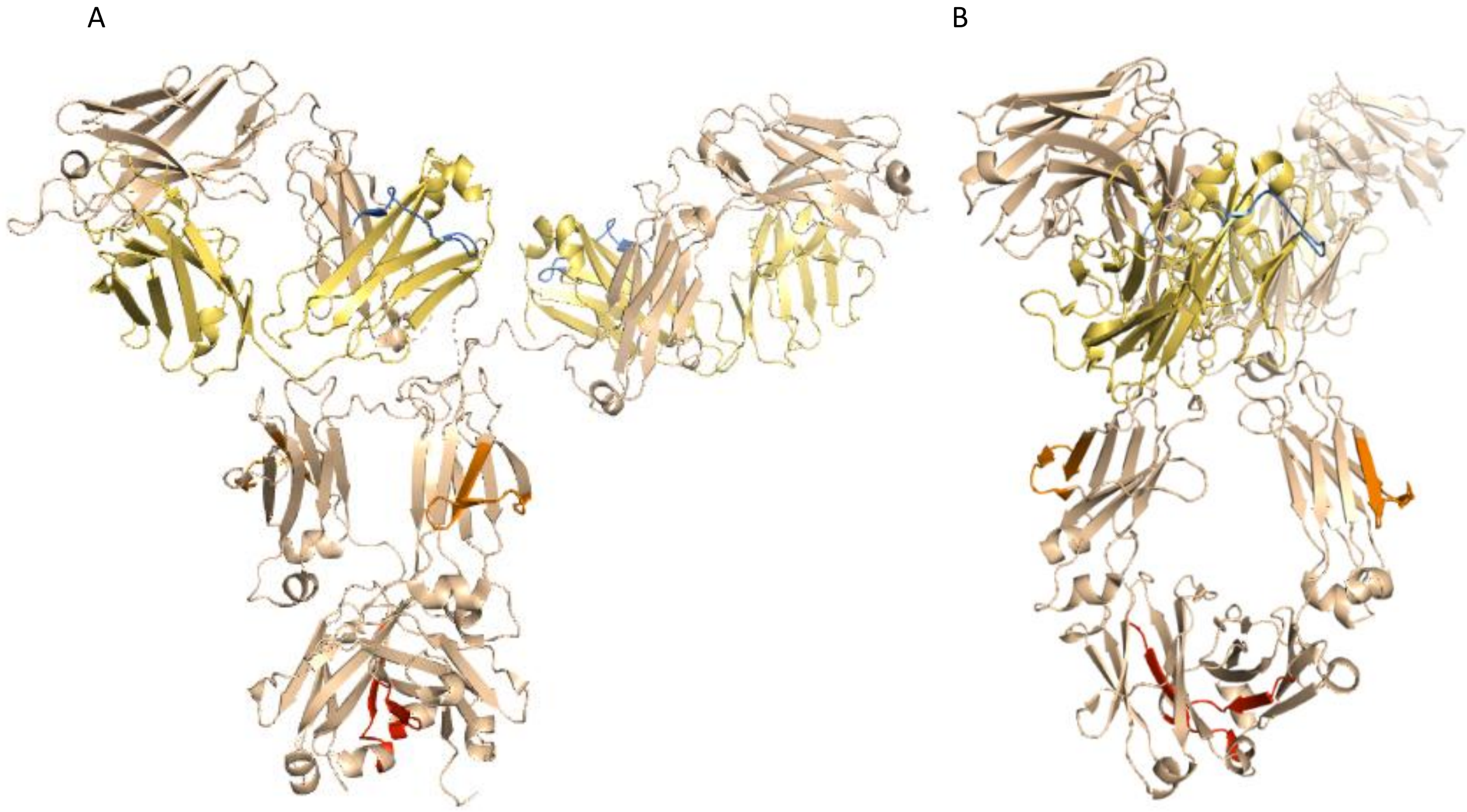

PyMOL IgG, reproduced crystallised structure of human IgG (1HZH);[2] γ heavy chain (ivory), k light chain (light yellow); pep_1_VDN (blue), pep_3_FNW (orange), pep_4_EPQ (red).

Fig. S2.

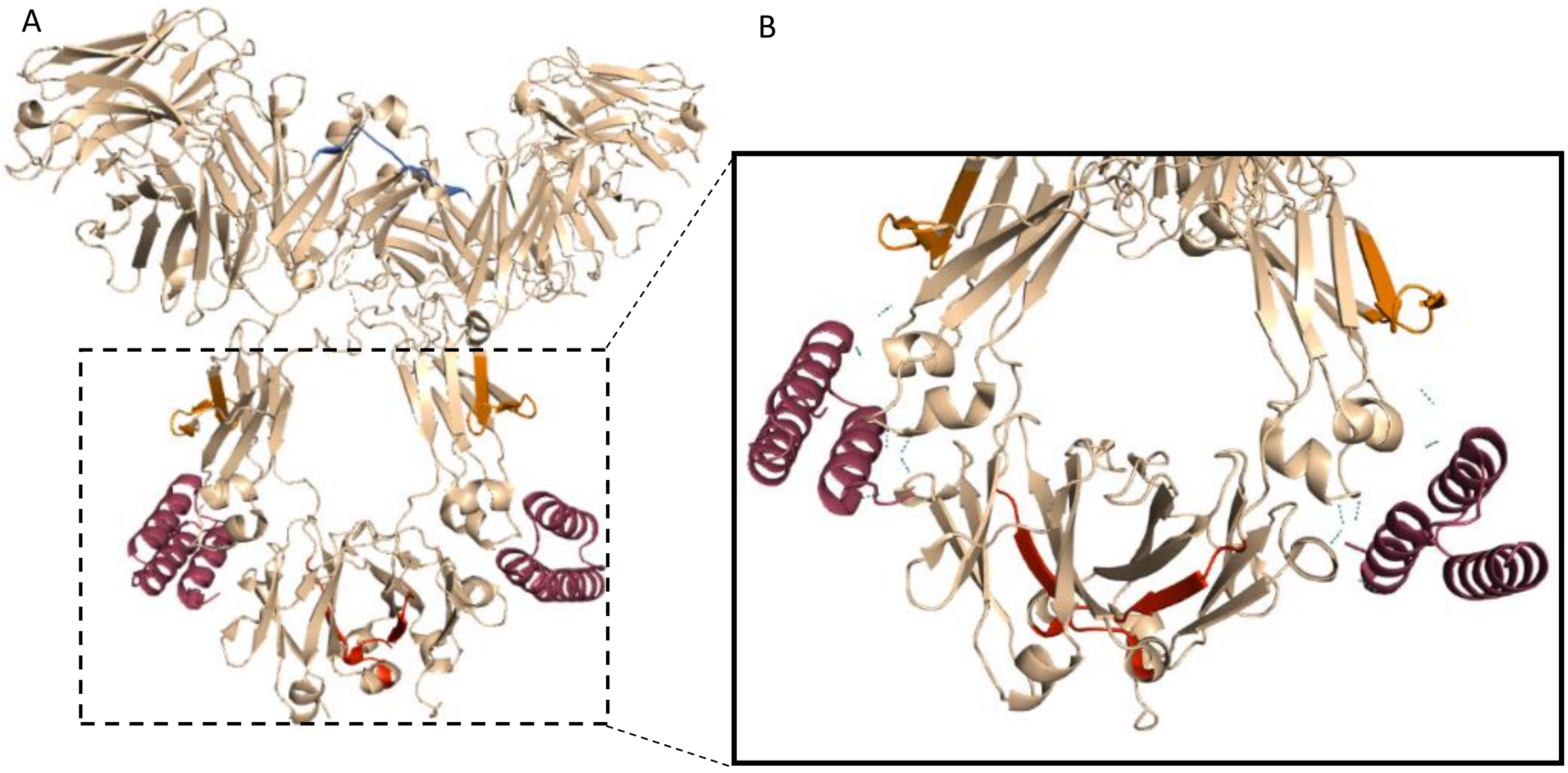

PyMOL alignment between IgG Fc bound to 3 helix of the B-domain from protA (5U4Y)[4] and human IgG (1HZH)[5] (A); magnified image of protA showing polar contacts (B).

Fig. S3.

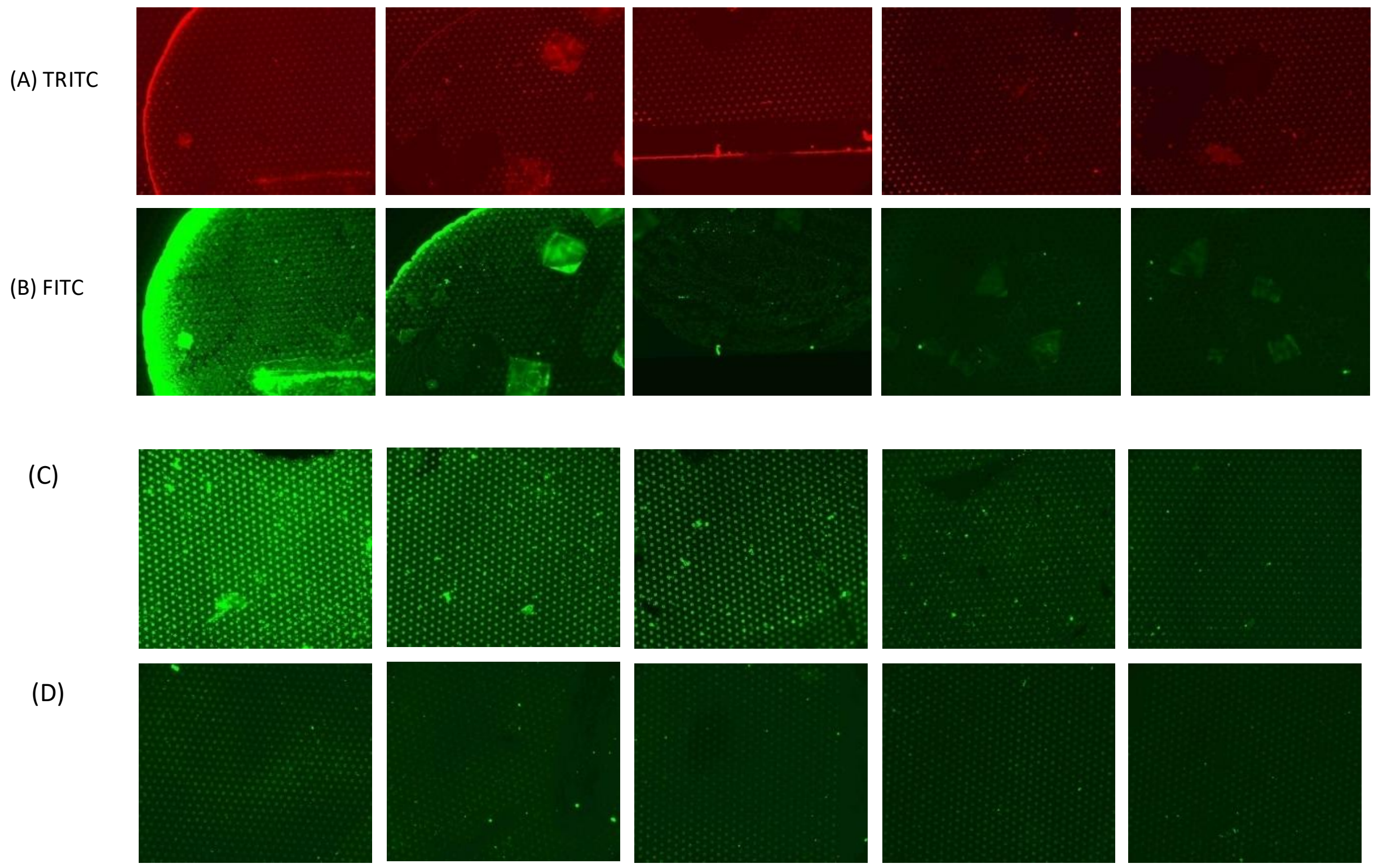

Fluorescent images, Left to right, decreasing concentrations of drop casted IgG_D488 [0.01 – 100 µg/mL]; image recorded in TRITC (A); and FITC (B).

Fluorescent images of IgG/IgG_D488. Left to right, decreasing concentrations of immobilised of IgG [0.01 – 100 µg/mL], IgG_D488 [10 µg/mL] (C); IgG_D488 [1 µg/mL] (D);

Fig. S4.

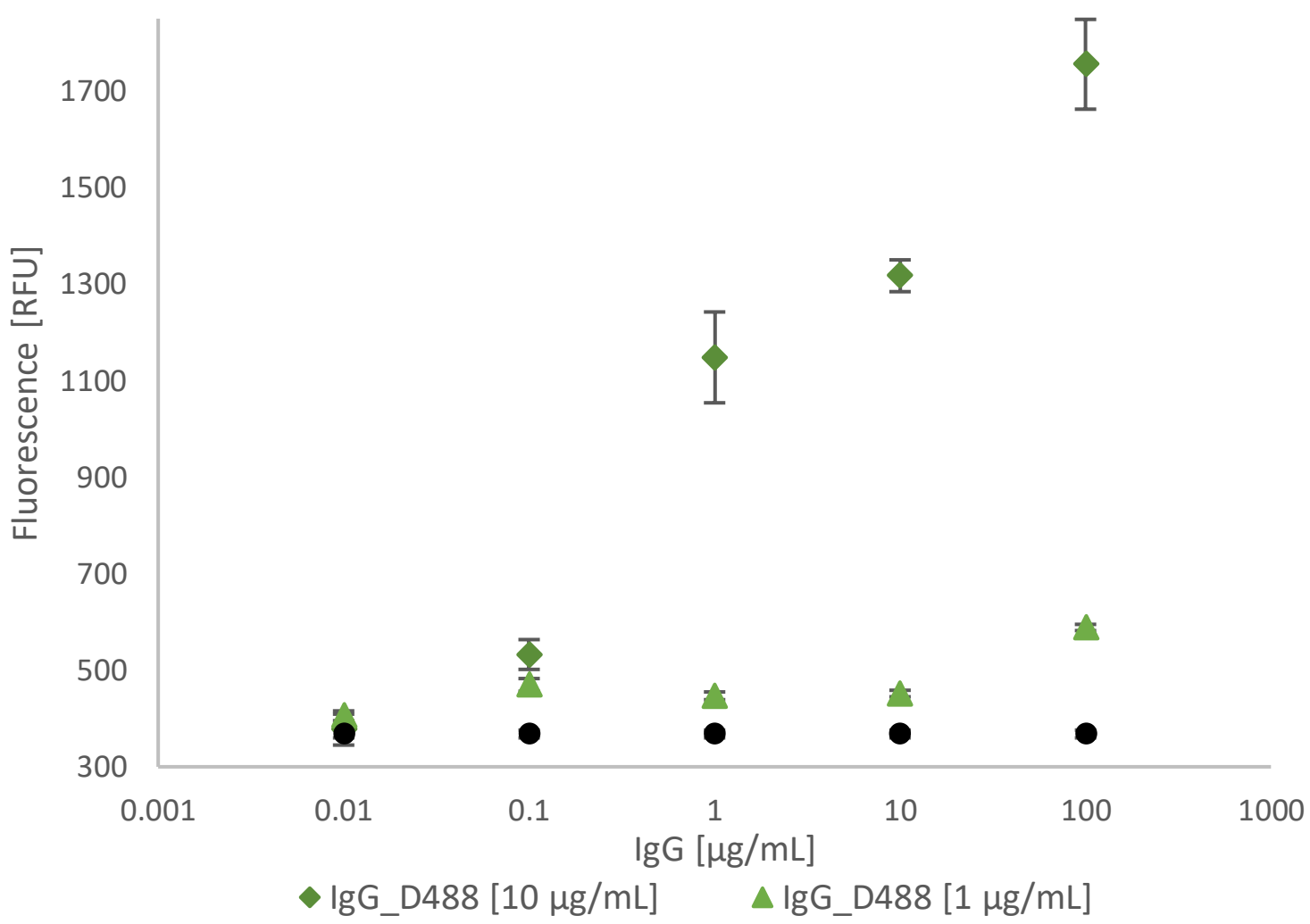

Fluorescent values. Immobilised IgG [0.01 – 100 µg/mL], IgG_D488 10 µg/mL (dark green); IgG_D488 1 µg/mL (light green); control in the absence of IgG (black).

Fig. S5.

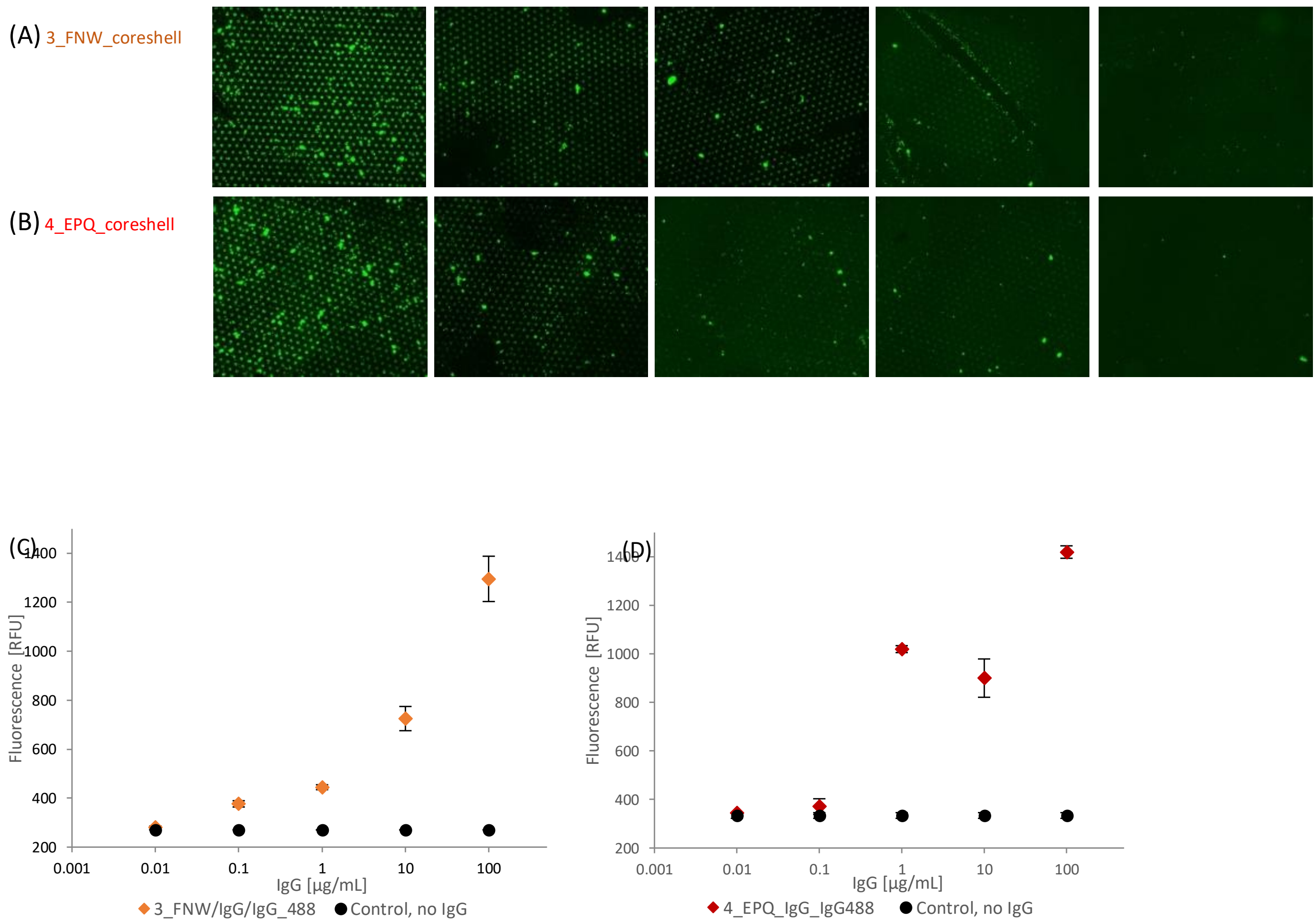

Fluorescent images and values, left to right, decreasing concentrations of IgG [0.01 – 100 µg/mL], IgG_D488 [10 µg/mL]. 3_FNW_coreshell (A); 4_EPQ_coreshell (B). Fluorescent values, IgG [0.01 – 100 µg/mL], IgG_D488 [10 µg/mL]; 3_FNW_coreshell (C); 4_EPQ_coreshell (D).

Fig. S6

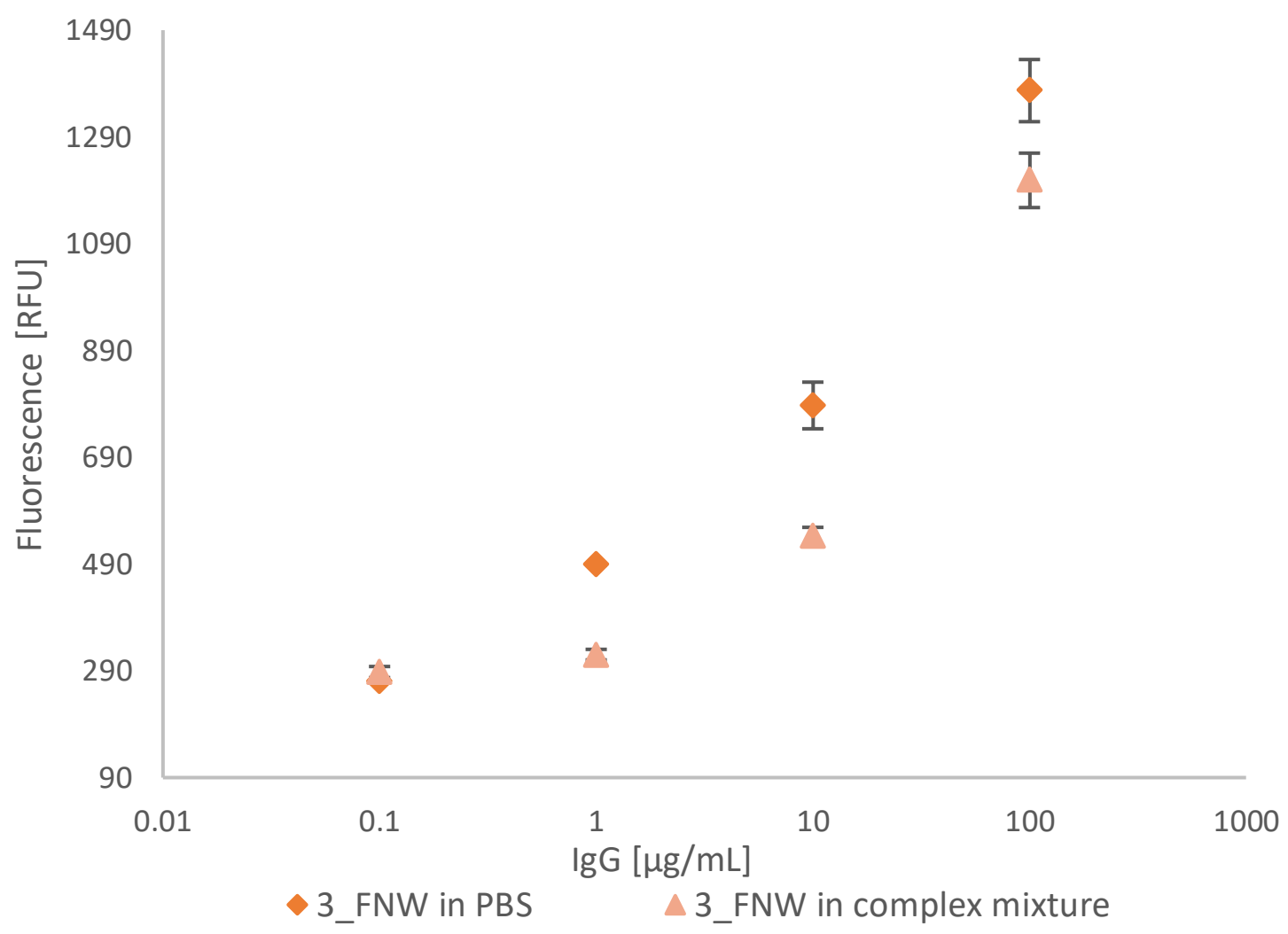

Fluorescent values, 3_FNW_coreshell, IgG [0.001 – 100 µg/mL], IgG_D488 [10 µg/mL] tested in PBS (dark orange); binding in complex mixture (light orange).

Fig S7.

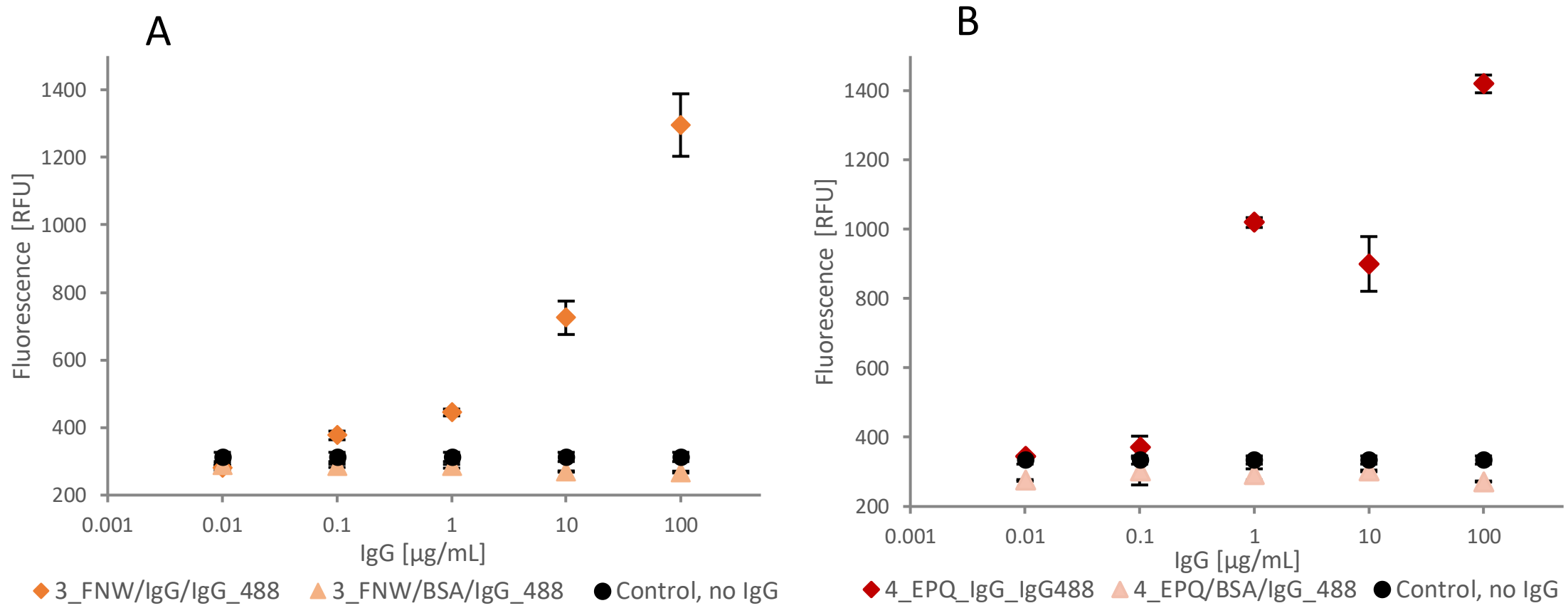

Fluorescent values, cross-reactivity tests using BSA instead of IgG concentration range [0.01-100 µg/mL], IgG_D488 [10 µg/mL]. 3_FNW_coreshell/IgG/IgG_D488 vs 3_FNQ_coreshell/BSA/IgG_D488 and control (A); 4_EPQ_coreshell/IgG/IgG_D488 vs 4_EPQ_coreshell/BSA/IgG_D488 and control (B).

Fig. S8

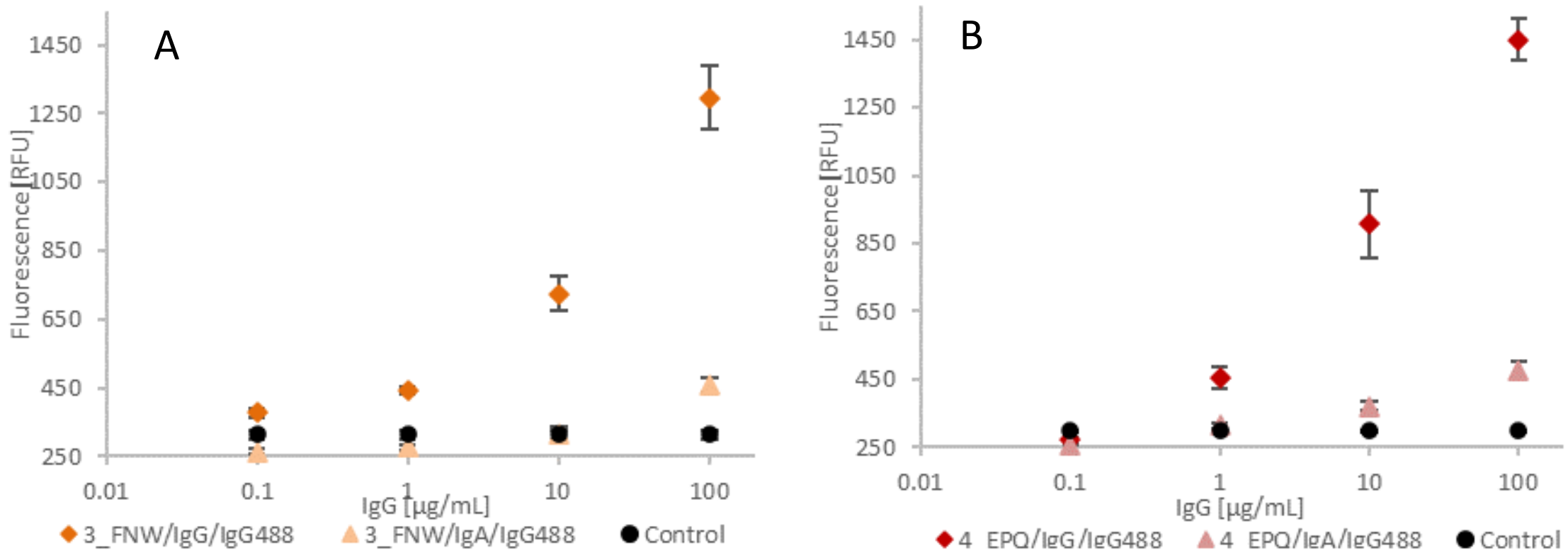

Fluorescent values, 3_FNW_coreshell nanoMIPs (A) and 4_EPQ_coreshell nanoMIPs (B) immobilized and tested alternatively for IgG [0.01 – 100 µg/mL], (dark orange), and IgA [0.01 – 100 µg/mL] (light orange), control in the absence of IgG (black), IgG_D488 [10 µg/mL].

Fig. S9

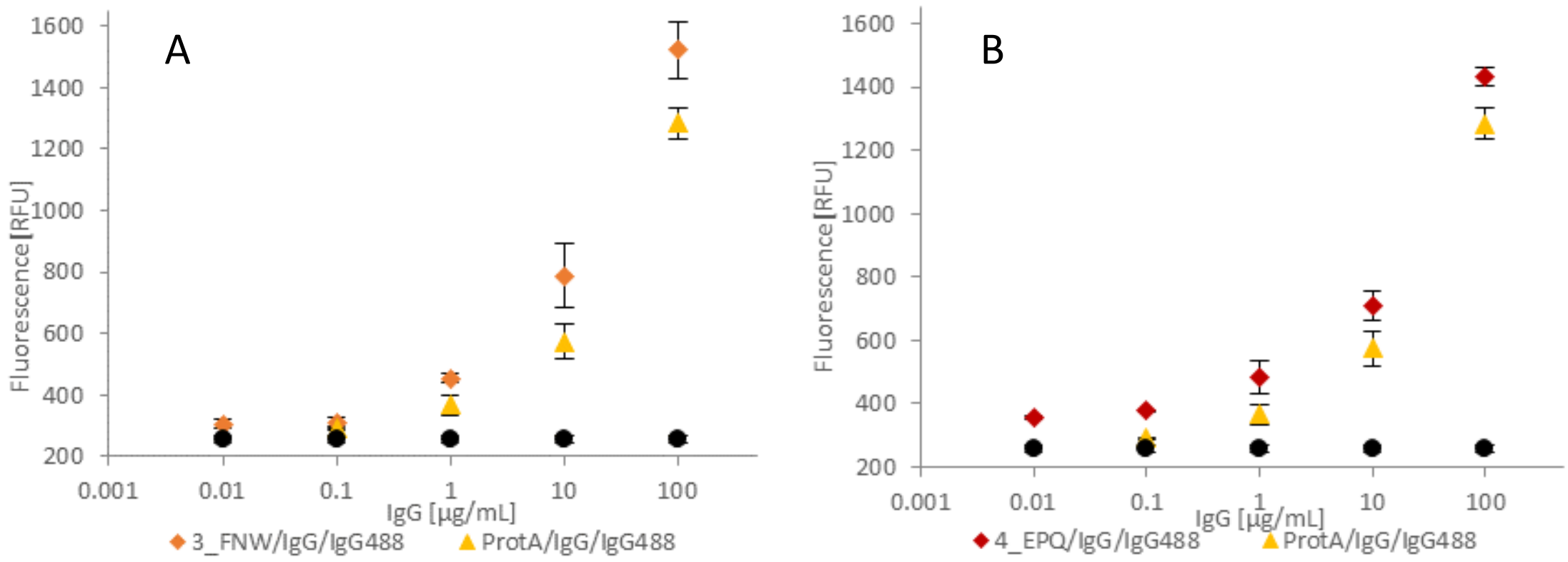

Fluorescent values, 3_FNW_coreshell nanoMIPs (orange, A) and 4_EPQ_coreshell nanoMIPs (red, B) immobilised and tested alternatively for IgG [0.01 – 100 µg/mL], and protA [0.01 – 100 µg/mL] (yellow), IgG_D488 [10 µg/mL].

Fig. S10

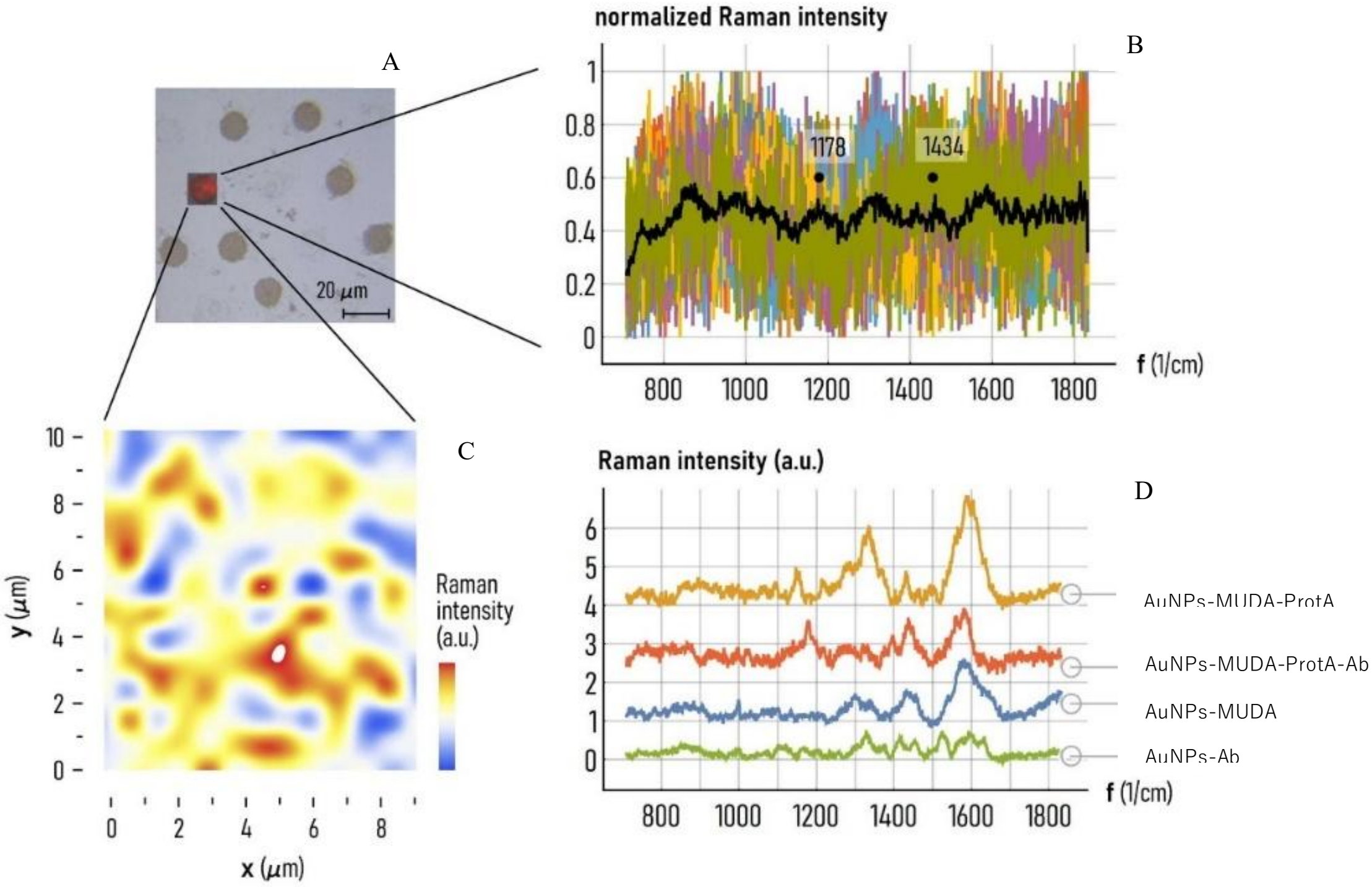

Optical image of gold array template, analysed by Raman spectroscopy (A); Raman spectra collected during a mapping analysis; The averaged spectrum (black) evidence the two significative peaks at points 1178 cm^{-1} (the histidine and tyrosine signal) and 1434 cm^{-1} (CH_2 bending peak) (B). Map of distribution of the Raman intensity for the 1178 cm^{-1} peak (C); Raman spectra of the different functionalisation steps (D).

Fig. S11

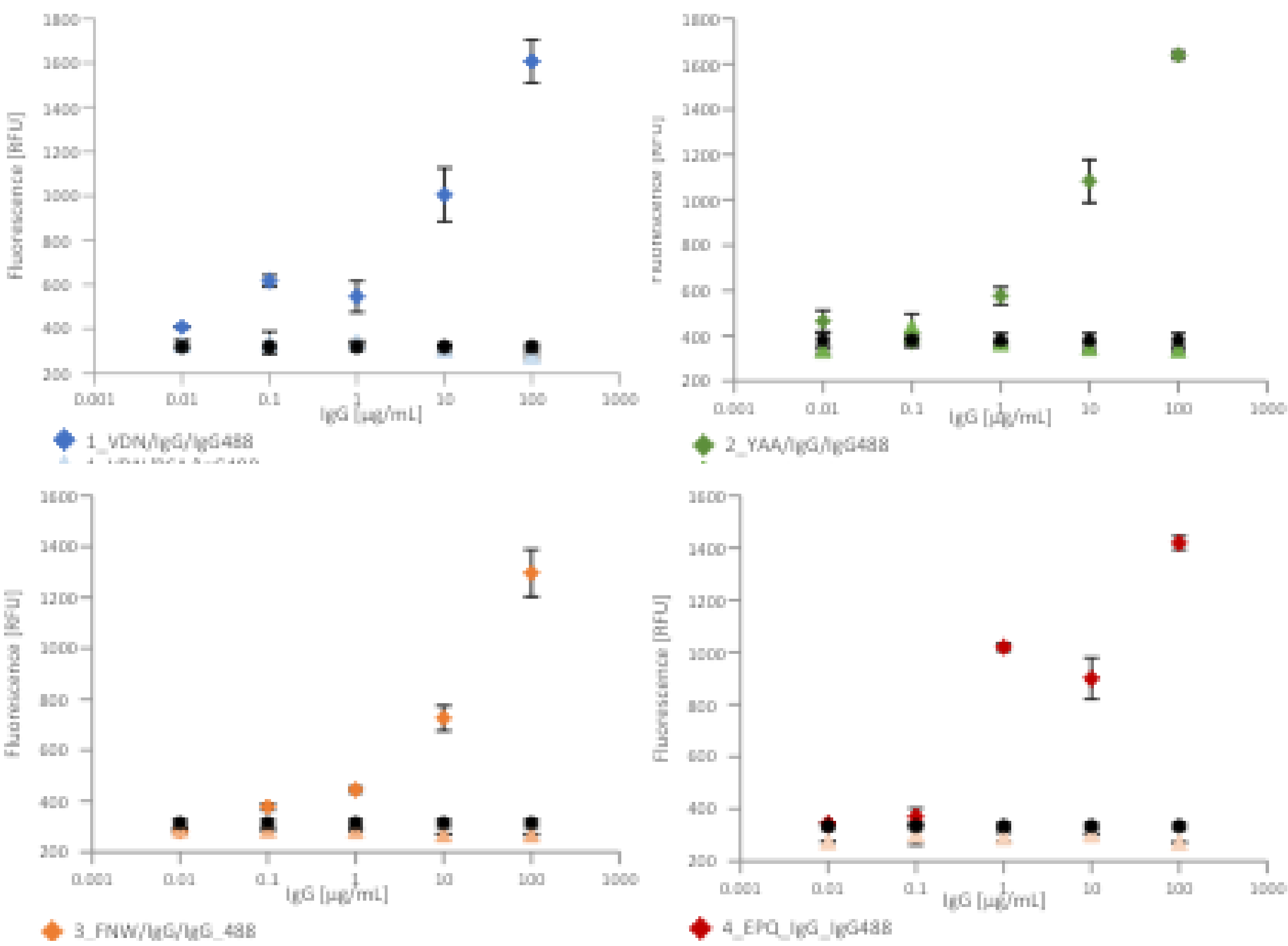

Fluorescent values, cross-reactivity tests using BSA instead of IgG concentration range [0.01-100 µg/mL], IgG_D488 [10 µg/mL]. 1_VDN_coreshell/IgG/IgG_D488 vs 1_VDN_coreshell/BSA/IgG_D488 and control (a); 2_YAA_coreshell/IgG/IgG_D488 vs 2_YAA_coreshell/BSA/IgG_D488 and control (b); 3_FNW_coreshell/IgG/IgG_D488 vs 3_FNQ_coreshell/BSA/IgG_D488 and control (c); 4_EPQ_coreshell/IgG/IgG_D488 vs 4_EPQ_coreshell/BSA/IgG_D488 and control (d).

Fig. S12

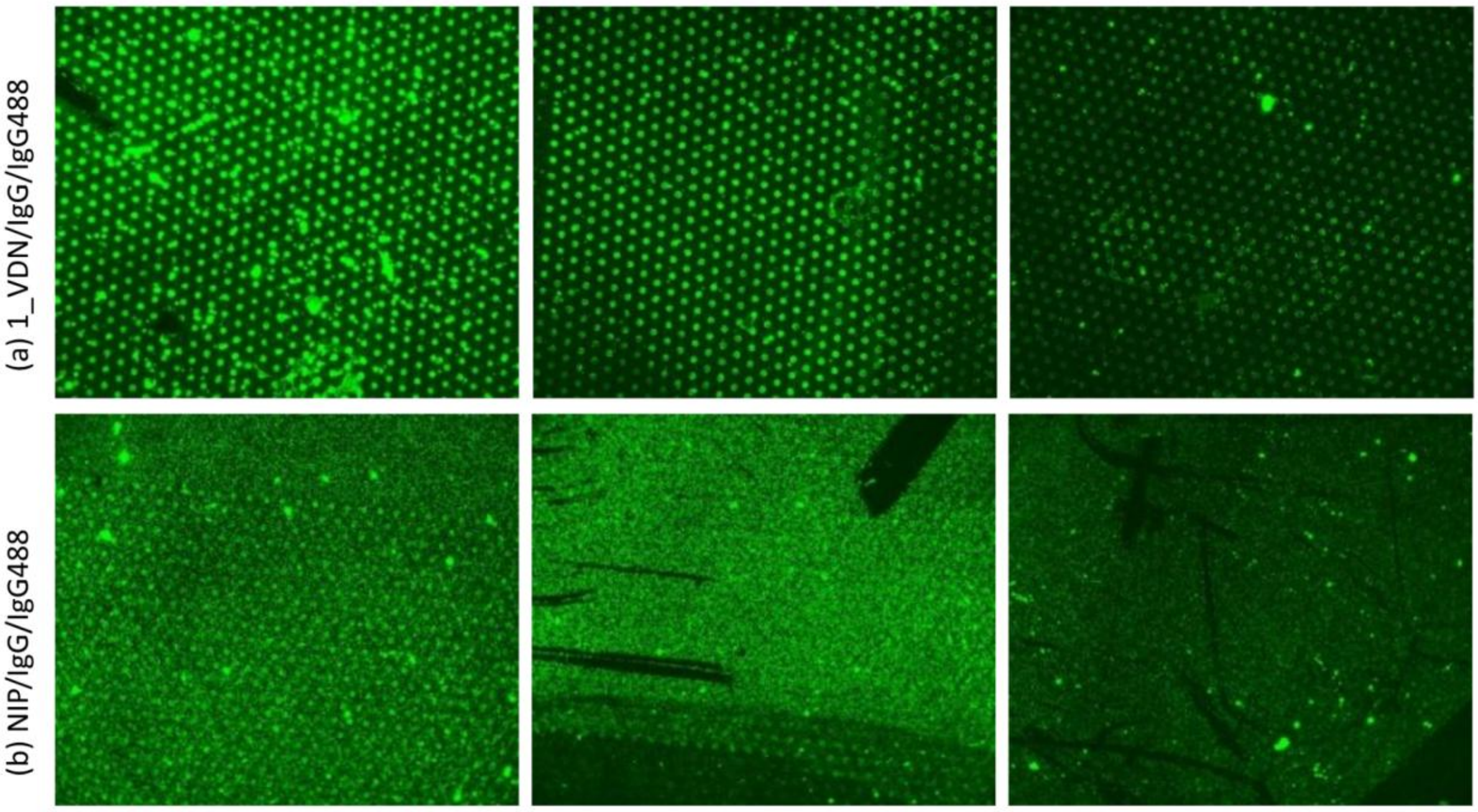

Fluorescent images, left to right, decreasing concentrations of IgG [1 – 100 μg/mL], IgG_D488 [10 μg/mL]; 1_VDN_coreshell (a); control-MIP (b).

Fig. S13

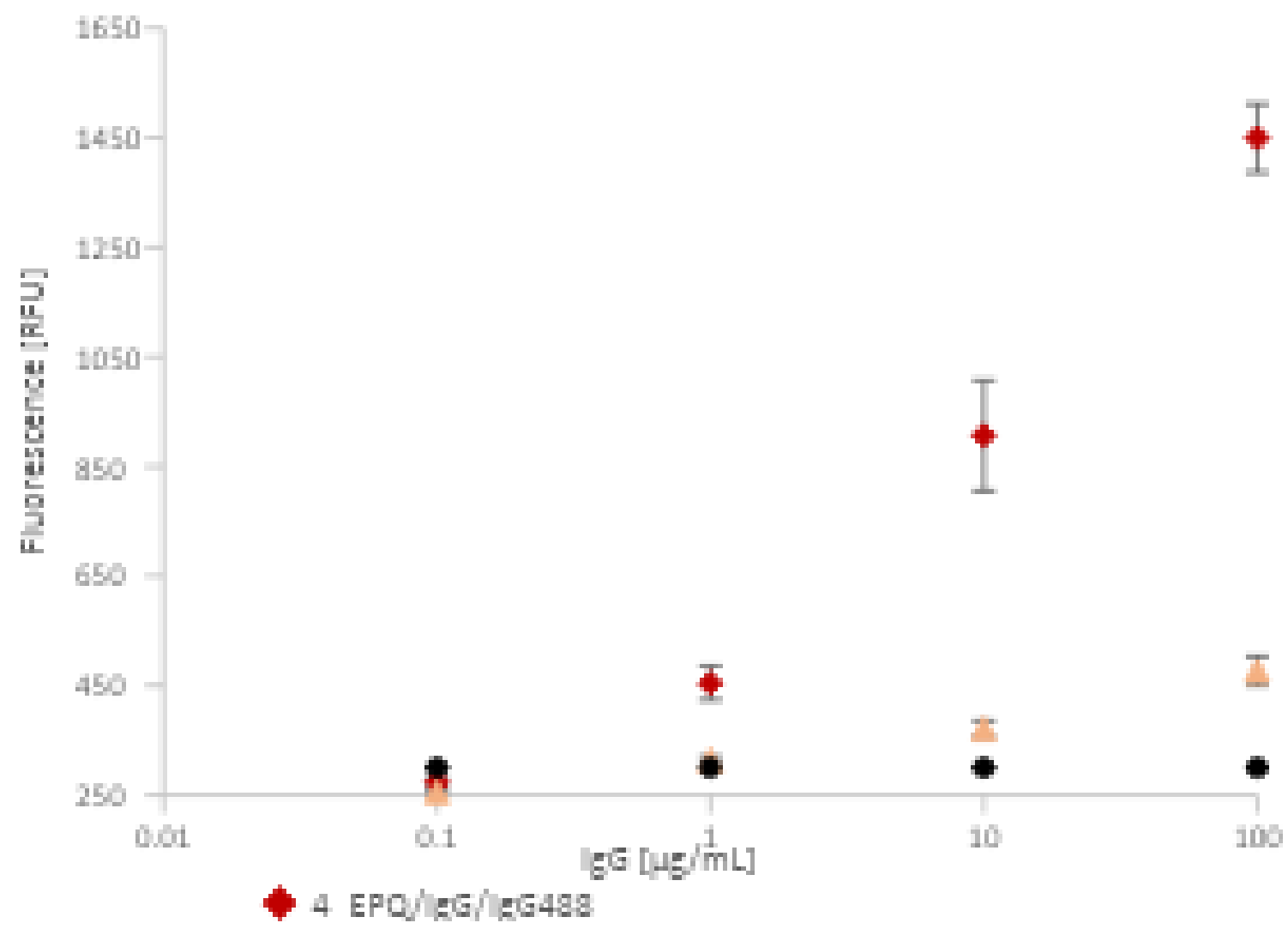

Fluorescent values, 4_EPQ_coreshell nanoMIPs immobilised and tested alternatively for IgG [0.01 – 100 µg/mL], (red), and IgA [0.01 – 100 µg/mL] (pink), control in the absence of IgG (black), IgG_D488 [10 µg/mL].

Fig. S14

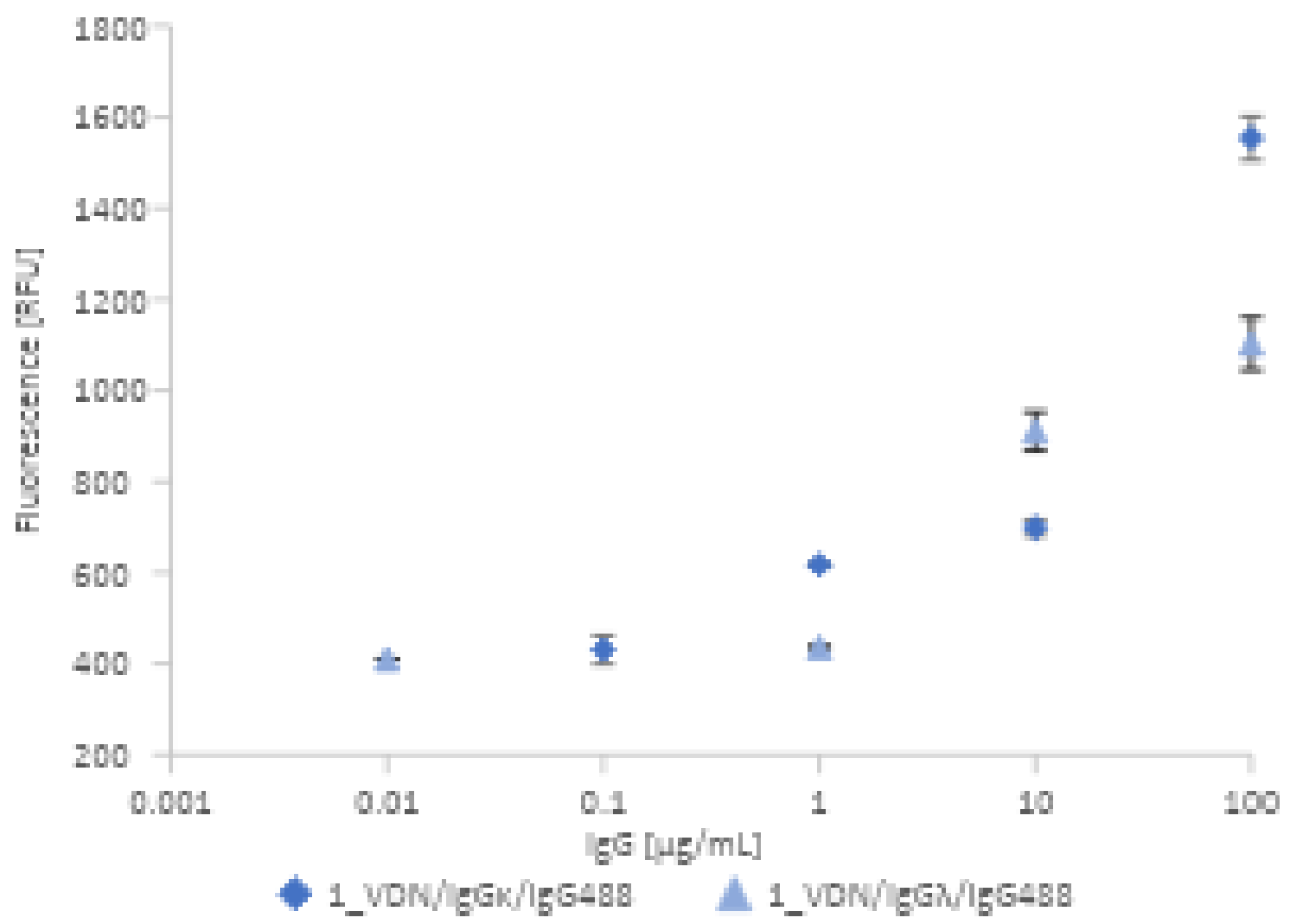

Fluorescent values, 1_VDN_coreshell nanoMIPs immobilised and tested alternatively for IgGκ [0.01 – 100 µg/mL], (dark blue), and IgGλ [0.01 – 100 µg/mL] (light blue), IgG_D488 [10 µg/mL].

Fig. S15

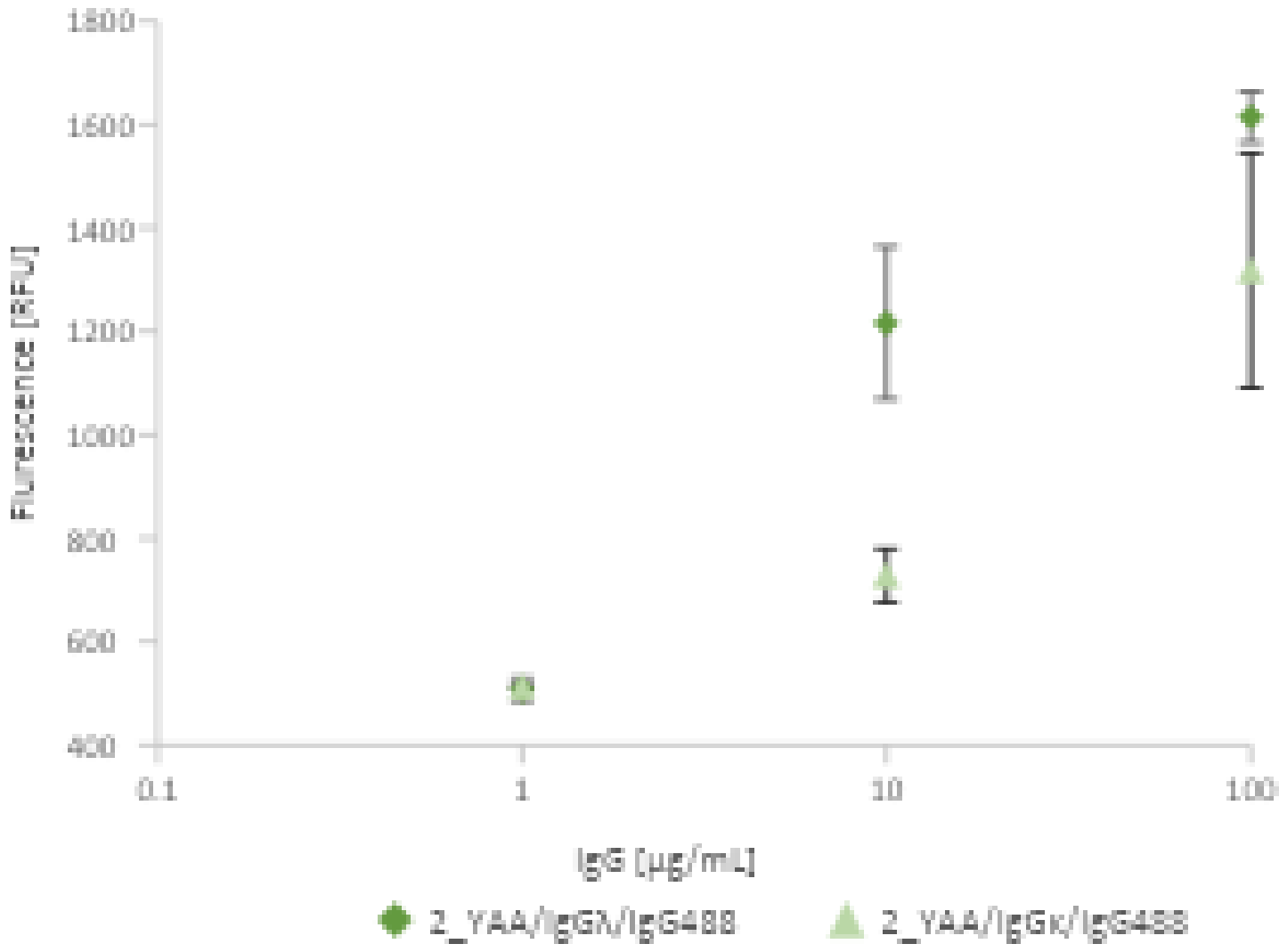

Fluorescent values, 2_YAA_coreshell nanoMIPs immobilised and tested alternatively for IgGκ [0.01 – 100 µg/mL], (dark green), and IgGλ [0.01 – 100 µg/mL] (light green), IgG_D488 [10 µg/mL].

Fig. S16

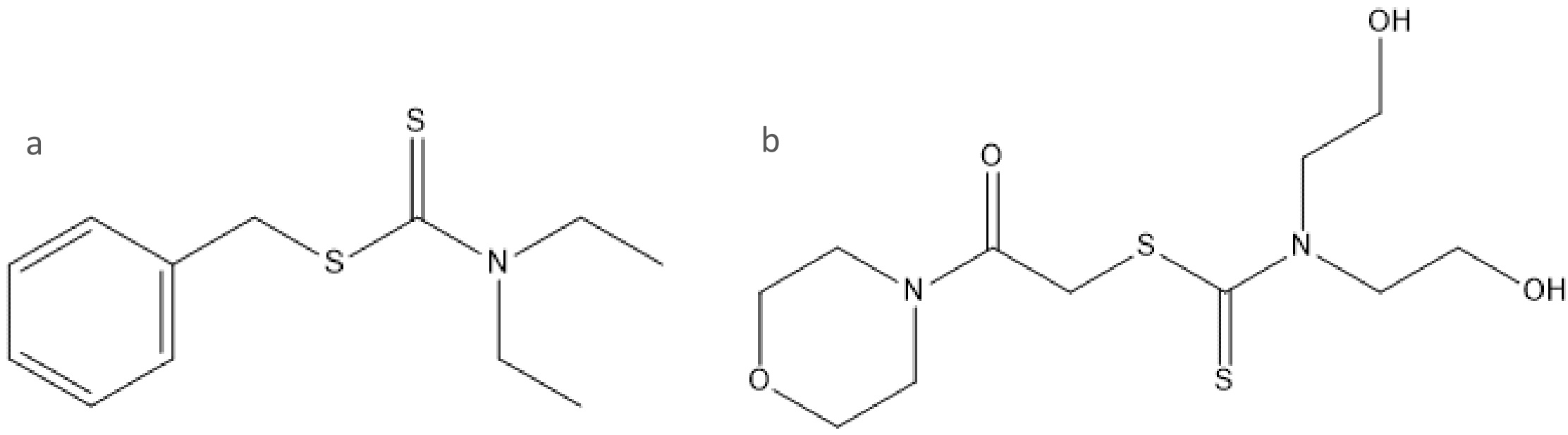

Structure of organic soluble iniferter, N,N-diethyldithiocarbamic acid benzyl ester (a); water soluble iniferter, 2-morpholino-2-oxoethyl bis(2-hydroxyethyl)carbamodithioate (b).

Fig. S17 Fluorescent images, left to right, decreasing concentrations of IgG [0.01 – 100 µg/mL], IgG_D488 [10 µg/mL].1_VDN_coreshell (a); 2_YAA_coreshell (b); 3_FNW_coreshell (c); 4_EPQ_coreshell (d).

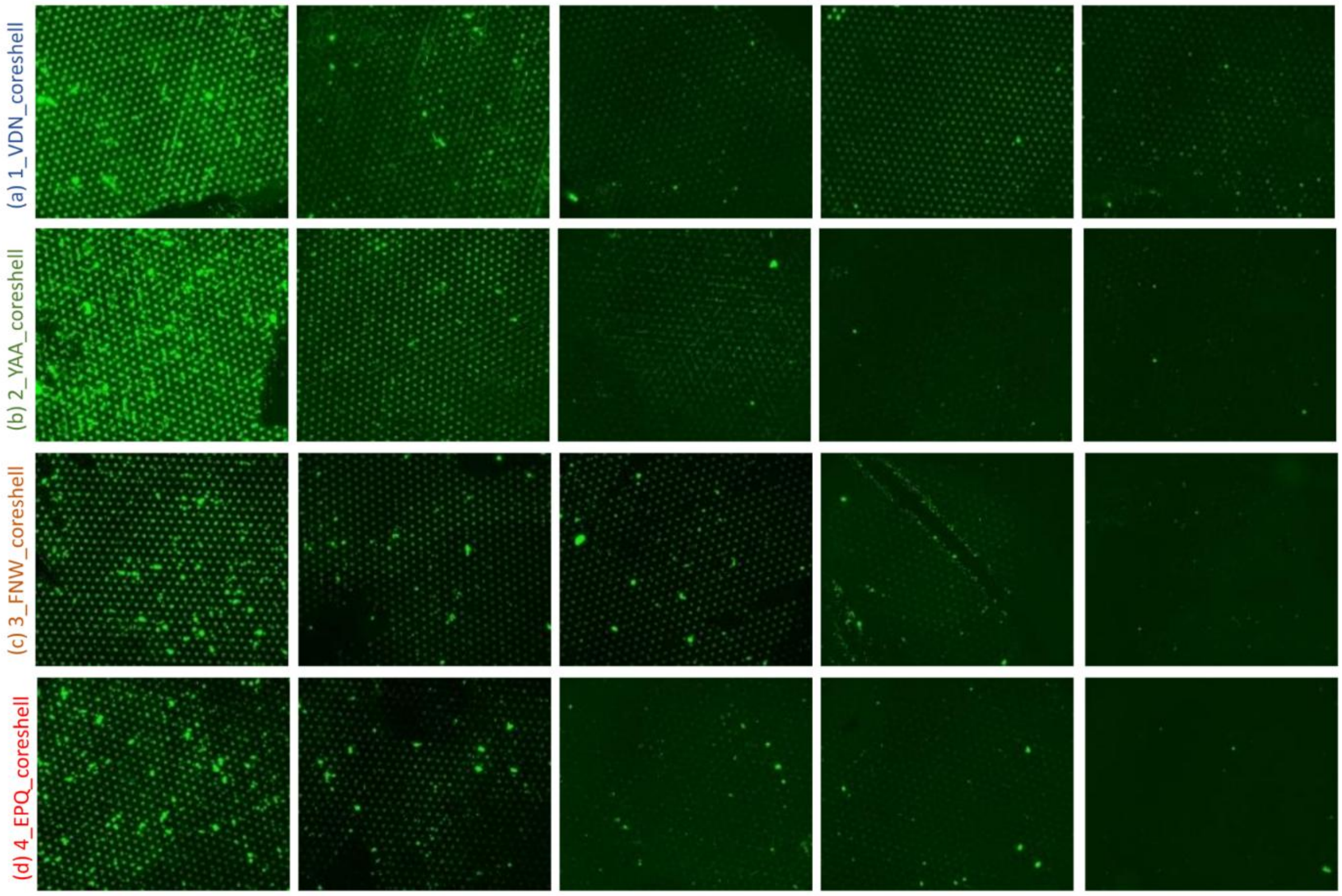

Fig. S18

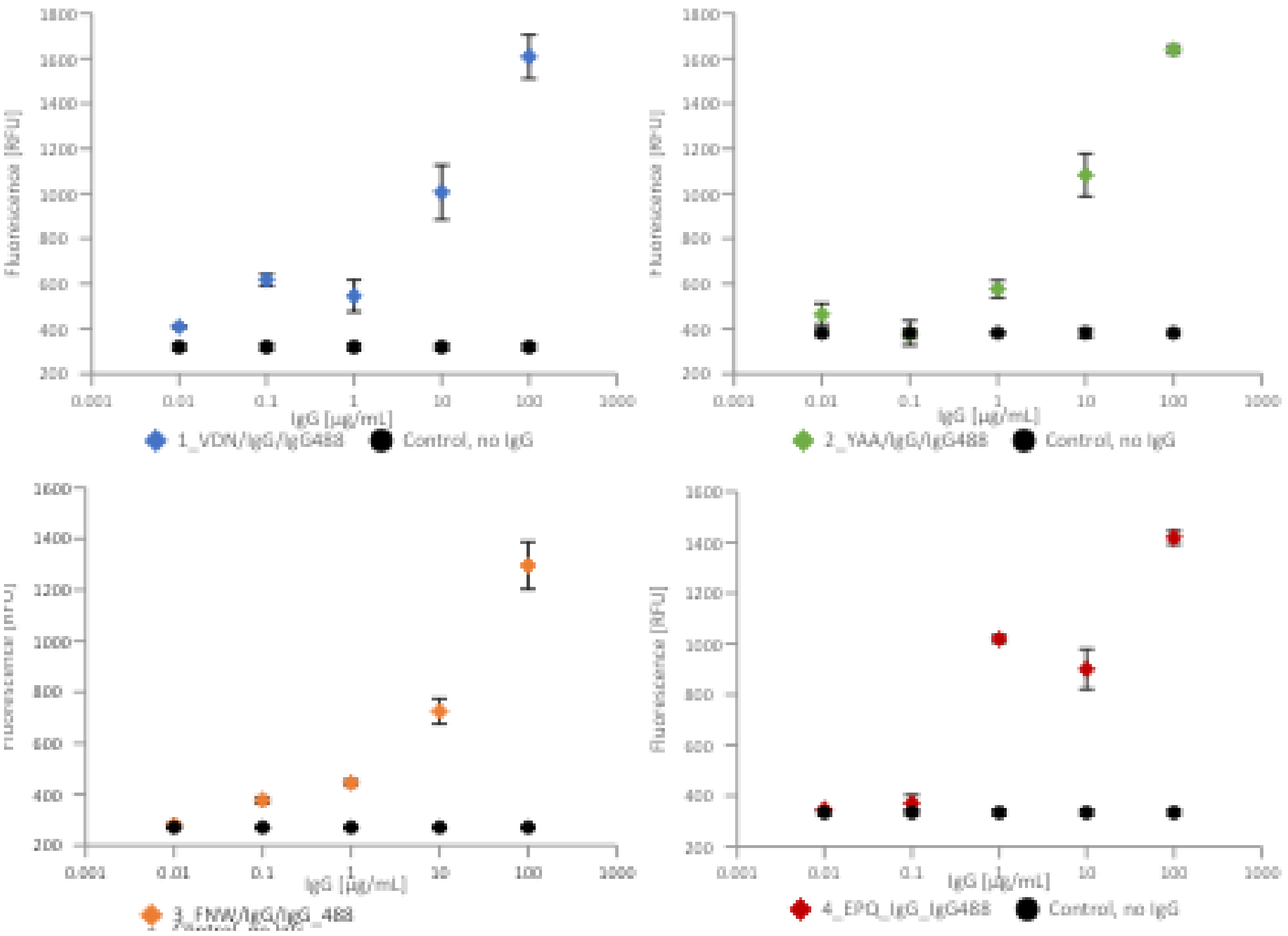

Fluorescent values, IgG [0.01 – 100 µg/mL], IgG_D488 [10 µg/mL]; 1_VDN_coreshell (a); 2_YAA_coreshell (b); 3_FNW_coreshell (c); 4_EPQ_coreshell (d);

Fig. S19

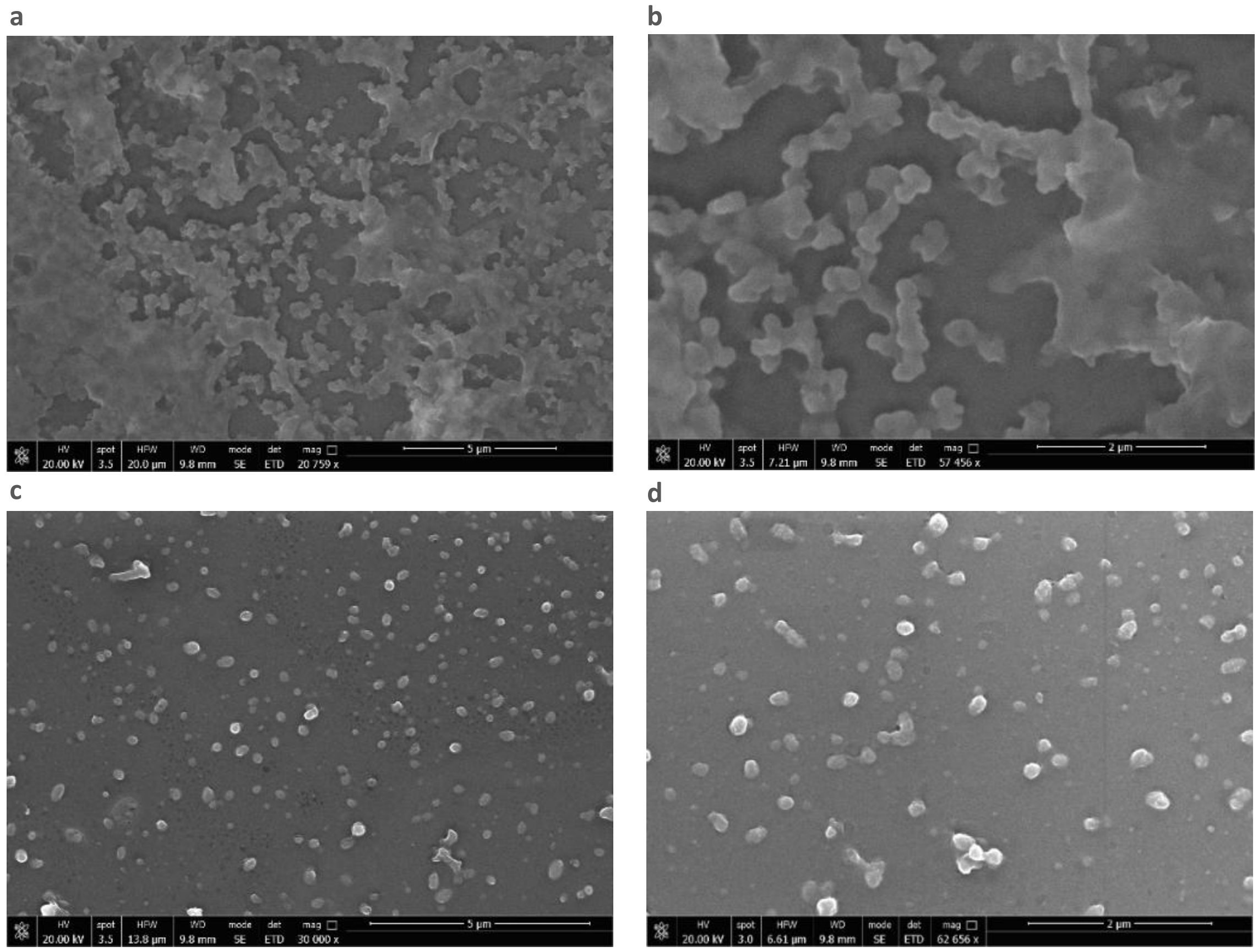

SEM images. SEM images of 1_VDN_coreshell nanoMIPs; scale bar 5 µm (a); 1_VDN_coreshell scale bar 2 µm (b); 1_VDN_stndrd nanoMIPs scale bar 5 µm (c); scale bar 2 µm (d).

Fig. S20

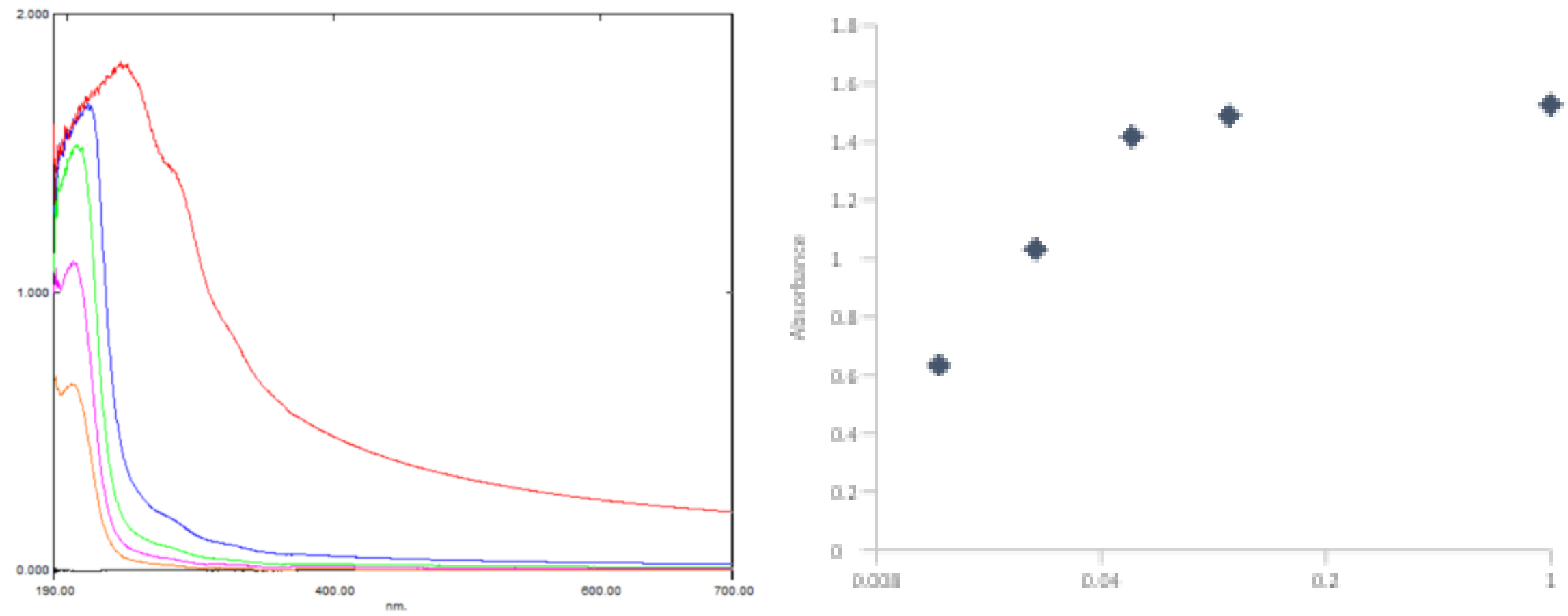

Dilution. Dilution of nanoMIPs UV analysis, peak read at 197 nM.

Fig. S21

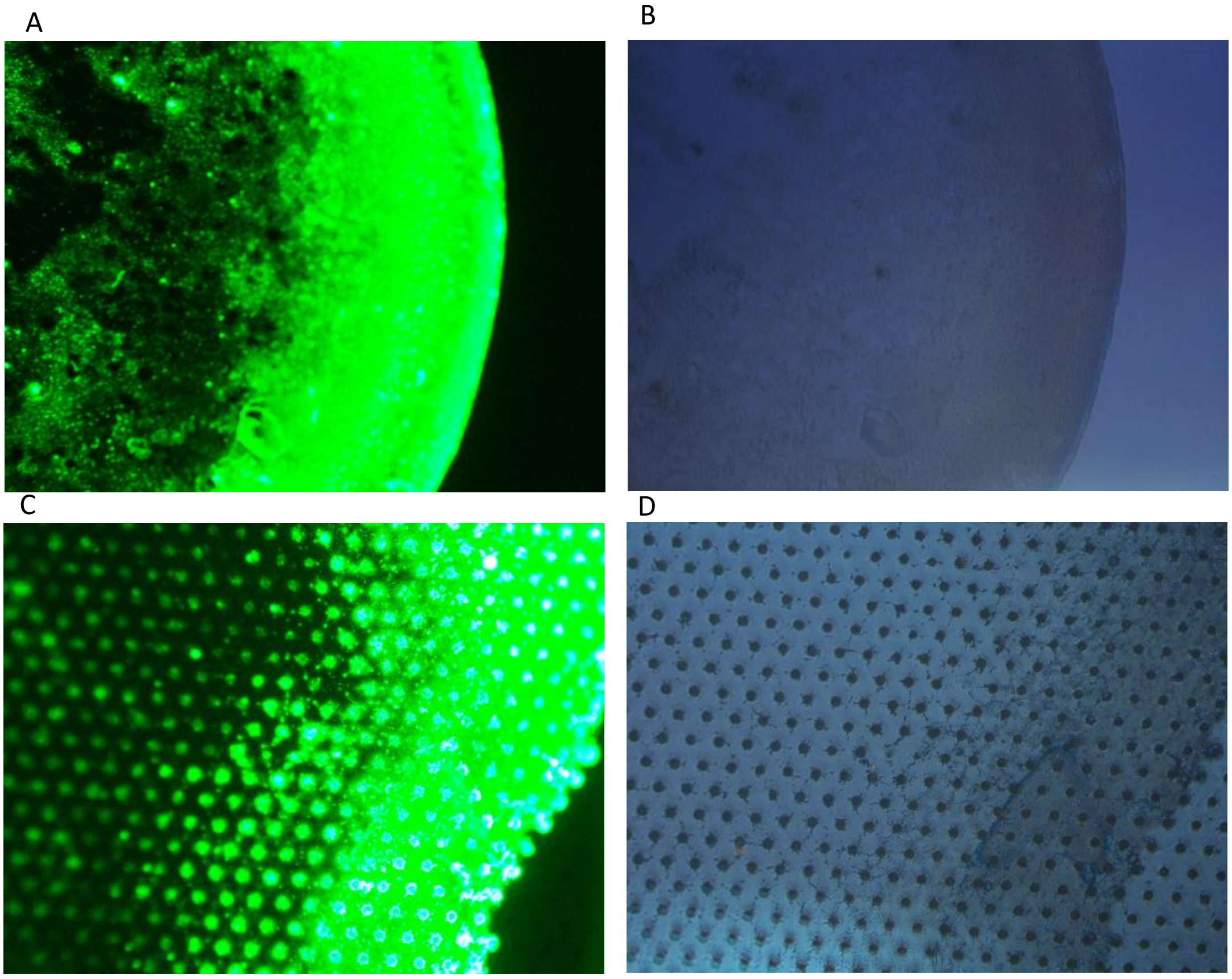

IgG_D488 drop-casted and dried on silicon surface, fluorescently excited (FITC) (A); bright field (B); IgG_D488 drop-casted and dried on silicon surface patterned with gold nanoclusters, fluorescently excited (C); bright field (D).

Fig. S22

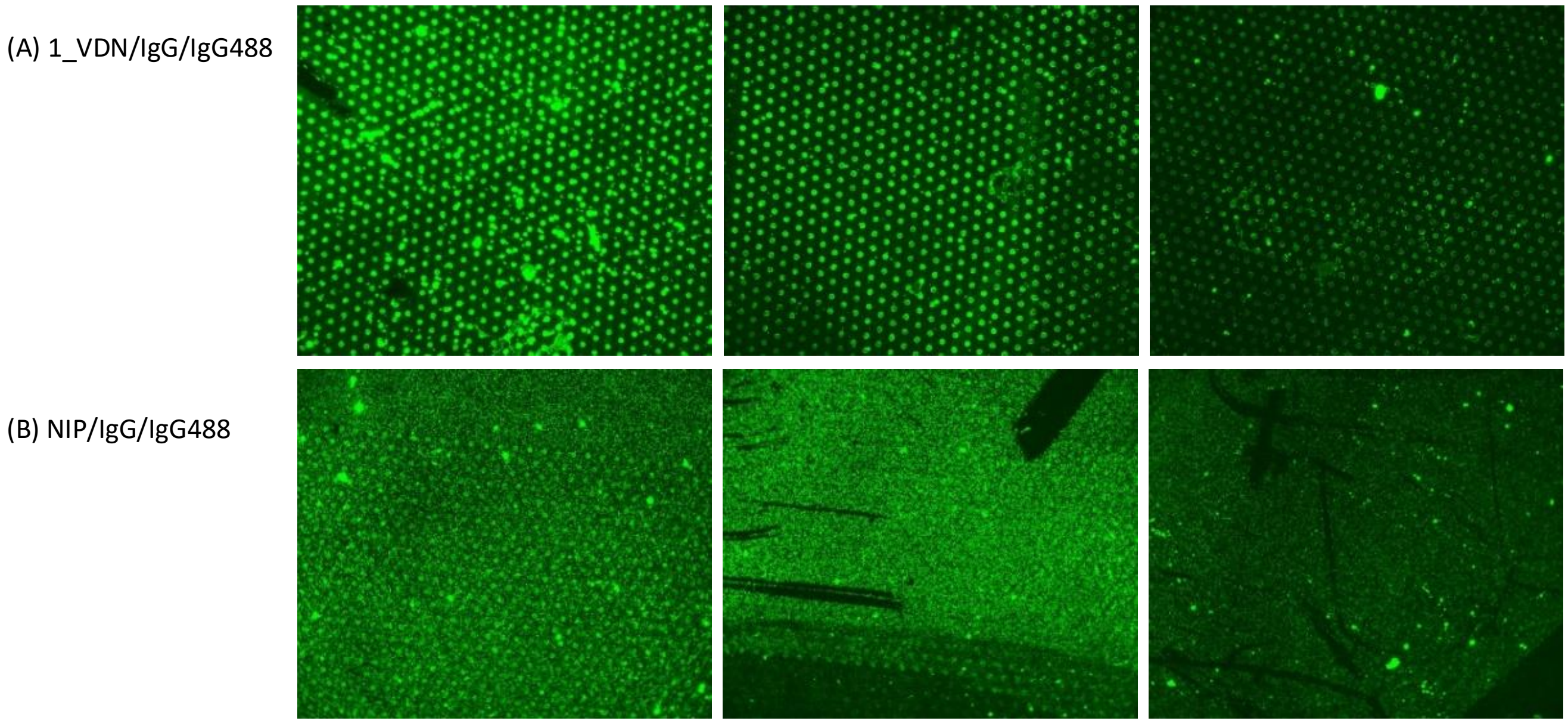

Fluorescent images, left to right, decreasing concentrations of IgG [1 – 100 µg/mL], IgG_D488 [10 µg/mL]; 1_VDN_coreshell (A); control-MIP (B).

Fig. S23

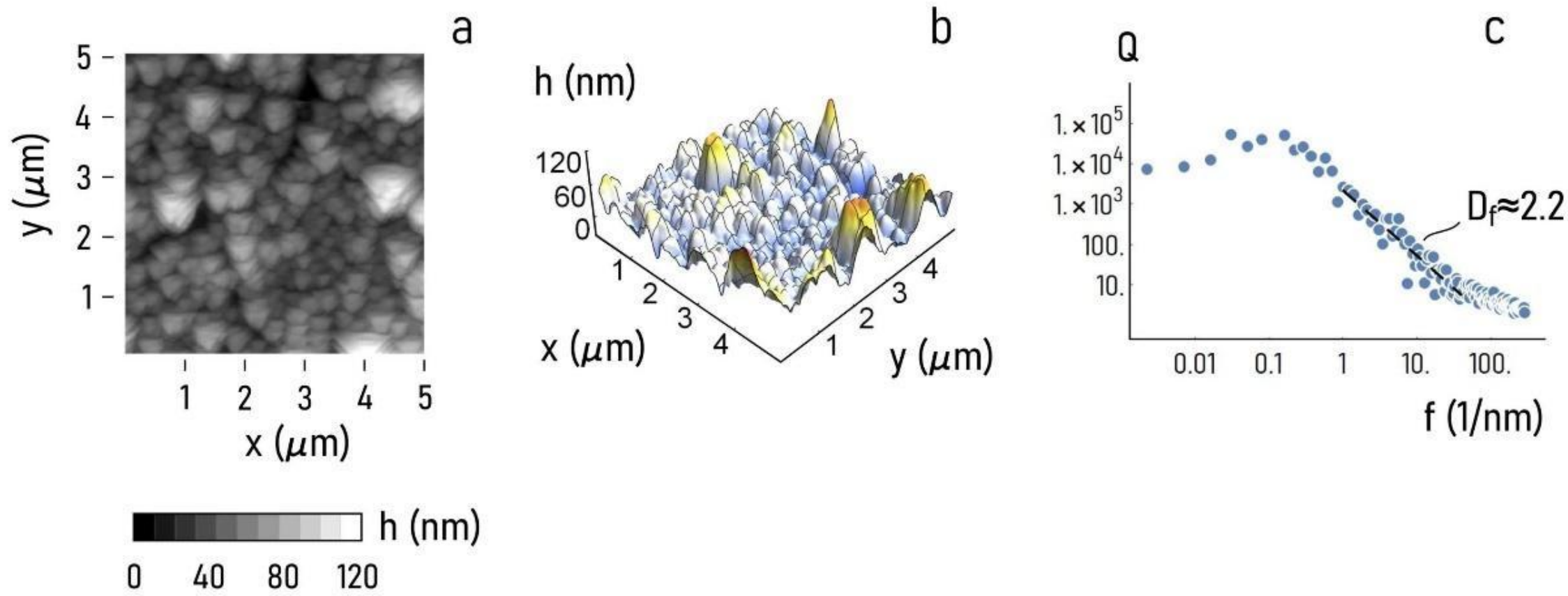

Atomic Force Microscopy (AFM) image; surface density plot (A) and 3D (B); power spectrum density function extracted from the AFM scan (C).

Table S1.

	Epitope identified	*Position on IgG*	*Position on the crystallised structure*	*UniProtKB code*
3_FNW	FNWYVDGVEVHNAK	*γ heavy chain constant region*	288-301	1HZH[54]
4_EPQ	EPQVYTLPPSR	*γ heavy chain constant region*	366-376	1HZH[54]

Summary of the IgG epitopes identified via Epitope Discovery and their position on the crystallised structure of IgG according to Protein Data Bank database.

Table S2.

Sample name:	*Epitope identified*	*Position on IgG*	*Identity in the allignment*
3_FNW_coreshell	FNWYVDGVEVHNAK	*γ heavy chain constant region*	100 % ϒ, 0 % μ, 0% α
4_EPQ_coreshell	EPQVYTLPPSR	*γ heavy chain constant region*	100 % ϒ, 70 % μ, 62 % α

Summary of alignment between each peptide selected for imprinting and each class of Ig, alignment run using protein blast (https://blast.ncbi.nlm.nih.gov/).

Table S3.

Short name	*density (g/mL)*	*MW (g/mol)*	*TOT volume (L)*	*Silane V (mL)*	*mass (g)*	*n (mol)*	*M (mol/L)*	*v/v %*
APTES	0.946	221.37	0.02	0.600	0.5676	0.0026	0.1282	3.00%
AEAPTES	1.028	222.36	0.02	0.586	0.5701	0.0026	0.1282	2.93%
AHAMTES	0.928	292.495	0.02	0.696	0.7500	0.0026	0.1282	3.48%

Silanes used to investigate stability

Table S4

**Sample name:**	**Epitope identified**	**Position on IgG**	**Identity in the allignment**
**1_VDN_coreshell**	VDNALQSGNSQ	*κ light chain constant region*	100 % κ, 0 % λ (human)
**2_YAA_coreshell**	YAASSYLSLTPEQW	*λ light chain constant region*	100 % λ, 0% κ (human)
**3_FNW_coreshell**	FNWYVDGVEVHNAK	*γ heavy chain constant region*	100 % ϒ, 0 % μ, 0% α
**4_EPQ_coreshell**	EPQVYTLPPSR	*γ heavy chain constant region*	100 % ϒ, 70 % μ, 62 % α

Summary of alignment between each peptide selected for imprinting and each class of Ig, alignment run using protein blast (https://blast.ncbi.nlm.nih.gov/).

Table S5 .

**Peptide name:**	**Sequence**	**Immobilisation method**	**Number of residues**
**Pep1_VDN**	VDNALQSGNSQ	GA	11
**Pep2_YAA**	YAASSYLSLTPEQW	GA	14
**Pep3_FNW**	**-CG**FNWYVDGVEVHNA**K**	SIA	14 (+2)
**Pep4_EPQ**	**-CG**EPQVYTLPPS**R**	SIA	11 (+2)

Summary of peptides information relative to sequence, immobilisation method, and number of residues

Table S6

**Sample name**	**Synthesis type**	**Size by DLS (nm)**	**Concentration by UV (mg/mL)**
1_VDN_coreshell	Core-shell	59.61 ± 0.75	0.58
2_YAA_coreshell	Core-shell	57.90 ± 5.10	0.59
3_FNW_coreshell	Core-shell	90.17 ± 1.07	0.86
4_EPQ_coreshell	Core-shell	83.47 ± 1.41	0.72

Summary of nanoMIPs imprinted for peptides characterisation by size and concentration.

References

1. F. Canfarotta, A. Poma, A. Guerreiro and S. Piletsky, Nature Protocols, 2016, 11, 443, https://doi.org/10.1038/nprot.2016.030.

2. Y. Fuchs, O. Soppera and K. Haupt, Anal Chim Acta, 2012, 717, 7-20, https://doi.org/10.1016/j.aca.2011.12.026.

3. Heidt, B.; Rogosic, R.; Lowdon, J. W.; Desmond-Kennedy, M.; Jurgaityte, K.; Ferrer Orri, J.; Kronshorst, Y.; Mendez, S.; Polyakova, E.; Rice, H. T., Biomimetic Bacterial Identification Platform Based on Thermal Transport Analysis Through Surface Imprinted Polymers: From Proof of Principle to Proof of Application. *physica status solidi (a)* 2019**,** *216* (12), 1800688, https://doi.org/10.1002/pssa.201800688.

4. Ultsch, M.; Braisted, A.; Maun, H. R.; Eigenbrot, C., 3-2-1: Structural insights from stepwise shrinkage of a three-helix Fc-binding domain to a single helix. *Protein Engineering, Design and Selection* 2017**,** *30* (9), 619-625, https://doi.org/10.1093/protein/gzx029.

5. Saphire, E. O.; Parren, P. W. H. I.; Pantophlet, R.; Zwick, M. B.; Morris, G. M.; Rudd, P. M.; Dwek, R. A.; Stanfield, R. L.; Burton, D. R.; Wilson, I. A., Crystal Structure of a Neutralizing Human IgG Against HIV-1: A Template for Vaccine Design. *Science* 2001, *293* (5532), 1155-1159, https://doi.org/10.1093/protein/gzx029.
